# Glycan Profile Analysis of Engineered Trastuzumab with Rationally Added Glycosylation Sequons Presents Significantly Increased Glycan Complexity

**DOI:** 10.3390/pharmaceutics13111747

**Published:** 2021-10-20

**Authors:** Esteban Cruz, Vicki Sifniotis, Zeynep Sumer-Bayraktar, Mouhamad Reslan, Lorna Wilkinson-White, Stuart Cordwell, Veysel Kayser

**Affiliations:** 1 Sydney School of Pharmacy, Faculty of Medicine and Health, The University of Sydney, Sydney, NSW 2006, Australia; ecru7298@uni.sydney.edu.au (E.C.); vsif0221@uni.sydney.edu.au (V.S.); mres7064@uni.sydney.edu.au (M.R.); 2 School of Life and Environmental Sciences, Faculty of Medicine and Health, The University of Sydney, Sydney, NSW 2006, Australia; zeynep.sumerbayraktar@sydney.edu.au (Z.S.-B.); stuart.cordwell@sydney.edu.au (S.C.); 3 Sydney Analytical, Core Research Facilities, The University of Sydney, Sydney, NSW 2006, Australia; lorna.white@sydney.edu.au

**Keywords:** protein aggregation, therapeutic monoclonal antibodies, Trastuzumab, glycosylation, biobetters

## Abstract

Protein aggregation constitutes a recurring complication in the manufacture and clinical use of therapeutic monoclonal antibodies (mAb) and mAb derivatives. Antibody aggregates can reduce production yield, cause immunogenic reactions, decrease the shelf-life of the pharmaceutical product and impair the capacity of the antibody monomer to bind to its cognate antigen. A common strategy to tackle protein aggregation involves the identification of surface-exposed aggregation-prone regions (APR) for replacement through protein engineering. It was shown that the insertion of *N*-glycosylation sequons on amino acids proximal to an aggregation-prone region can increase the physical stability of the protein by shielding the APR, thus preventing self-association of antibody monomers. We recently implemented this approach in the Fab region of full-size adalimumab and demonstrated that the thermodynamic stability of the Fab domain increases upon *N*-glycosite addition. Previous experimental data reported for this technique have lacked appropriate confirmation of glycan occupancy and structural characterization of the ensuing glycan profile. Herein, we mutated previously identified candidate positions on the Fab domain of Trastuzumab and employed tandem mass spectrometry to confirm attachment and obtain a detailed *N*-glycosylation profile of the mutants. The Trastuzumab glycomutants displayed a glycan profile with significantly higher structural heterogeneity compared to the HEK Trastuzumab antibody, which contains a single *N*-glycosylation site per heavy chain located in the CH2 domain of the Fc region. These findings suggest that Fab *N*-glycosites have higher accessibility to enzymes responsible for glycan maturation. Further, we have studied effects on additional glycosylation on protein stability via accelerated studies by following protein folding and aggregation propensities and observed that additional glycosylation indeed enhances physical stability and prevent protein aggregation. Our findings shed light into mAb glycobiology and potential implications in the application of this technique for the development of “biobetter” antibodies.

## 1. Introduction

The advent of therapeutic monoclonal antibodies (mAb) reshaped the pharmaceutical industry and has enabled a wide array of therapeutic avenues. Antibody therapeutics have dominated the market for the past two decades, featuring 7 mAbs or mAb-based therapeutics in the top-10 list of best-selling drugs in 2018; and are now implemented in the treatment of an extensive range of pathologies, including oncological, inflammatory, cardiovascular, neurodegenerative and infectious diseases [1,2,3,4]. Moreover, mAbs comprise the largest class of molecules undergoing clinical development, thus it is forecasted that their market size will grow further as their clinical applications expand [1,2,5].

The coming of age of mAbs as therapeutics entailed substantial efforts aimed at addressing pitfalls of first-generation molecules, most notably the immunogenicity caused by their non-human origin [6]. Yet, despite the myriad advancements in antibody technology, protein aggregation is a recurring complication that continues to hinder their manufacture and clinical properties [1,7]. Furthermore, the physicochemical factors determining thermodynamic and colloidal stability remain poorly understood. Aggregation can have a negative impact on the therapeutic efficacy of monoclonal antibodies by compromising their biological function, increasing clearance rates, and triggering immunogenic reactions. Moreover, the shelf-life of the pharmaceutical product can be severely reduced by the accelerated formation of protein aggregates [8,9].

Although it is believed that evolution has yielded improvements in the thermodynamic states of native antibodies relative to aggregated species, the manufacturing process and storage exposes recombinant therapeutic mAbs to a combination of “non-physiological” stress factors that hamper their physical and chemical stability [10,11]. These factors include high protein concentrations, mechanical stress, exposure to air–water interfaces, and variations in pH, temperature, and ionic strength. To tackle this issue, numerous strategies have been developed and are the subject of intense exploration. These approaches can be broadly classified into two main categories: those that enhance the intrinsic stability of the protein to prevent aggregation throughout the manufacturing process [12,13,14,15], and those that improve the formulation of the final product by the optimization of pH, ionic strength and the incorporation of stabilizing agents [16,17,18].

Excipients commonly used in protein stabilization include surfactants, sugars, amino acids, polymers, and other proteins. Recently, ionic liquids have emerged as promising additives in this context [18,19]. Whilst highly valuable, formulation strategies are limited in their applicability in that the amount of excipient added is restricted by several factors, including toxicity and increases in viscosity and tonicity [19]. Furthermore, optimizing formulation conditions for particular proteins is challenging, given the lack of understanding of aggregation mechanisms and how excipients impact these processes. In light of these limitations, strategies to improve the intrinsic physical stability of mAbs become highly valuable as a way to avoid physical degradation in the manufacturing process and/or decrease the necessity to incorporate excipients in the final formulation. This has been particularly relevant in recent times due to the growing trend to formulate antibody therapeutics as sub-cutaneous (SC) syringes for self-injection, wherein high protein concentrations are required to deliver effective doses in the limited injection volumes allowed by the SC route [1,20].

Approaches to improve intrinsic aggregation resistance rely on rational modifications of the antibody structure. It is widely believed that surface-exposed hydrophobic regions in proteins can lead to self-association and initiate the formation of larger aggregates [8,12]. To tackle this, in silico methods have been developed to screen and identify aggregation-prone regions (APR) [12,15,21,22]. Exposed hydrophobic amino acids in APRs can then be replaced with more hydrophilic or charged residues through mutagenesis [10,12,13]. Pepinsky et al. introduced an innovative variation to this approach, wherein the mutations pursued inserted an *N*-glycosylation sequon (Asn-X-Ser/Thr) in the Fab region of an anti-LINGO antibody, enabling the attachment of an additional glycan on the antibody molecule [23]. IgG Fc-glycans confer colloidal stability, and we have previously shown that aglycosylated mAbs aggregate at higher rates [24]. The substantial increments (50-fold) in solubility obtained with two such mutants were attributed to the large hydrodynamic size and hydrophilic character of the glycan, preventing self-association through steric hindrance. Later, Courtois et al. employed an advanced computational tool, called Spatial Aggregation Propensity (SAP), that performs full antibody molecular dynamic simulations to identify APRs in bevacizumab; and proposed a panel of residues that could be mutated to insert a glycosylation site where the glycan can shield an aggregation-prone region and deter protein-protein interactions [25]. In the latter study, significant enhancements were reported in accelerated stability studies with the glyco-engineered bevacizumab variants, reporting up to 3-fold improvements in monomer preservation. More recently, our group tested a panel of such mutants in adalimumab and demonstrated improvements in thermodynamic stability, reflected by increments in the second melting temperature (*T_m_*2) [26].

Altogether, the experimental results obtained with the glycosylation site insertion approach have highlighted the potential of this technique to improve shelf-life and pharmacokinetic profiles of approved therapeutic antibodies and mAbs in preclinical development. Notwithstanding, the aforementioned studies have lacked appropriate confirmation of glycan attachment; and more importantly, structural analysis of the glycans attached to the engineered site. The latter is essential in the context of regulatory approval and in elucidating the molecular mechanisms that drive improvements in stability. In view of the foregoing, we sought to ascertain differences in the glycosylation profiles of an in-house produced Trastuzumab antibody (non-mutant variant used as control, expressed in HEK-293F cells), compared to its glycoengineered mutants. Process conditions and host-cell choices have major effects on glycosylation profiles. Thus, Herceptin^®^ that is produced in CHO cells and our control Trastuzumab that is produced in HEK-293F cells have comparable but somewhat dissimilar glycosylation patterns.

Herein, we implemented several glycosite insertions in the Fab region of the blockbuster antibody Trastuzumab and characterized the full glycan profile of the Trastuzumab variants by glycan enzymatic release followed by LC–MS/MS analysis. In addition, we employed algorithms for prediction of successful glycan attachment to compare with experimental data to gain insight into structural features governing glycan transfer during translation. Furthermore, we evaluated the binding kinetics of the Trastuzumab variants to its biological target (HER2) and to Fc receptors involved in eliciting effector functions to assess potential effects on therapeutic efficacy.

## 2. Materials and Methods

Herceptin^®^ was a generous donation from Genentech (San Francisco, CA, USA). The pVITRO-1-Trastuzumab-IgG1/κ expression vector (containing Trastuzumab heavy- and light-chain genes) was a generous gift from Andrew Beavil (Addgene plasmid # 61883). The gWiz expression vector was purchased from Genlantis (San Diego, CA, USA). The primers utilized for mutagenesis and sequencing were acquired from Geneworks (Adelaide, Australia). The DpnI enzyme (R0176S) and the Phusion high-fidelity PCR kit (E0553S) were purchased from Genesearch (Gold Coast, Australia). Stellar^TM^ competent *E. coli* cells were purchased from Scientifix (Melbourne, Australia). Tryptone (LP0042B) was purchased from Thermo Fisher Scientific (Melbourne, Australia). Sodium chloride (S9888) and Kanamycin solution (K0254) were purchased from Sigma-Aldrich (Sydney, Australia). The miniprep (PLN70) and maxiprep (NA0310) kits were obtained from Sigma-Aldrich (Sydney, Australia).

Freestyle 293F cells were provided by Dr. Mario Torrado del Rey of Prof. Joel Mackay’s research group, School of Life and Environmental Sciences, The University of Sydney. FreeStyle^TM^ 293 media (12338018), RPMI media (21870-076), OptiPRO^TM^ serum free media (SFM) (12309050) and other reagents used for tissue culture were purchased from Life Technologies (Melbourne, Australia). The 25 kDa linear polyethylenimine (PEI) (23966-2) was purchased from BioScientific (Sydney, Australia). Disposable baffled tissue-culture flasks (CLS431405), glycine hydrochloride (G2879), and HiTrap^®^ Protein A HP affinity column (GE17-0403-01) were obtained from Sigma-Aldrich (Sydney, Australia). The Reprosil-Pur C18AQ (3 µm, 120 A) was purchased from Dr. Maisch (Amerbuch, Germany).

PBS tablets (09-2051-100), 1 M Tris-HCl pH 9 (BIOSD814), and all chemicals employed for SDS-PAGE were acquired from Astral Scientific (Sydney, Australia). Precision Plus Protein™ Dual Color Molecular Weight Marker (1610374) was obtained from Bio-Rad Laboratories (Sydney, Australia). Trypsin (T6567), Chymotrypsin (1141847001) were purchased from Sigma-Aldrich (Sydney, Australia). C18 ZipTip^®^ pipette tips (ZTC18S096) were obtained from Merck Millipore. PNGase F (P0708) was purchased from Genesearch (Gold Coast, Australia).

HEPES (54457), Tween 20 (P9416) and the HIS-tagged HER2 (SRP6405), FcγR2B (SRP6396), and FcγR3A (SRP6436) receptors were purchased from Sigma-Aldrich (Sydney, Australia). HIS-tagged FcγR1A (10256-H08H-5) were obtained from Life Technologies (Melbourne, Australia). The CM5 chips (29-1049-88), anti-HIS capture kit (28-9950-56), and amine coupling kit (BR-1000-50) were purchased from GE Healthcare (Sydney, Australia).

### 2.1. Cloning and Mutation of HEK Trastuzumab and Glycosylation Mutants

Cloning of the HEK Trastuzumab and the panel of glycosylation mutants into a mammalian expression vector was performed as described previously [26]. Briefly, a section of a bicistronic pVITRO-1-Trastuzumab-IgG1/κ expression vector containing a secretion signal and Tmab heavy-chain gene, and another section containing secretion signal and Tmab light kappa chain gene were amplified separately employing high fidelity PCR (Phusion High Fidelity PCR kit) with a 2-step PCR cycling method. These amplified sections were then inserted separately into a blank gWiz vector through restriction enzyme cloning to produce separate vectors for Trastuzumab heavy-chain (HC) and light-chain (LC) expression. Mutagenesis was performed through high fidelity inverse PCR to obtain mutated Trastuzumab expression vectors. Successful mutation was confirmed by sequencing. HEK-293F cells were co-transfected with heavy- and light-chain vectors for transient expression.

### 2.2. Expression and Purification of HEK Trastuzumab and Glycosylation Mutants

Expression and purification of HEK Trastuzumab and glycosylation mutants were performed as reported previously [26]. In brief, the Trastuzumab variants were expressed transiently in HEK-293F cells in suspension at 120 rpm, using Freestyle 293F media. The cell culture was maintained at 37 °C, 5% CO_2_. Transfection was performed at a cell density of 1 × 10^6^ cells/mL (60 mL cultures). Vector DNA and PEI in OptiPRO SFM were added to the cells in a 2:1 PEI/DNA *w*/*w* ratio. Total DNA added per 1 × 10^6^ cells was 1 µg (1:1 *w*/*w* HC to LC ratio). Following 24 h after transfection, the cultures were scaled up to 120 mL with Freestyle 293F media containing tryptone (0.5% *w*/*v* final concentration). At 48 h post transfection the cultures were further scaled up to 240 mL with Freestyle 293F media containing tryptone (0.5% *w*/*v* final concentration). The cell culture supernatant was harvested by centrifugation at day 8 following transfection and subsequently clarified by filtration (0.22 µm).

Purification of the secreted antibody was performed employing a HiTrap^®^ Protein A HP affinity column. PBS was used for the binding and washing steps. Elution of bound antibody was performed with 0.1 M glycine HCl pH 2.7 and the eluted fractions were immediately neutralized with 1 M Tris-HCl pH 9. Eluted fractions containing antibody were pooled and buffer exchanged to PBS using 50 kDa molecular weight cutoff (MWCO) centrifugal filters. Antibody concentrations were derived from absorbance readings at 280 nm using a molar extinction coefficient ε = 2.25 × 10^6^ M^−1^ cm^−1^.

### 2.3. LC–MS/MS Analysis of Tmab Variants

#### 2.3.1. Glycopeptide Analysis

Glycopeptide analysis through LC–MS/MS was performed as described previously [26]. Briefly, HEK Trastuzumab, Herceptin and Tmab mutants were run on a reducing 10% SDS-PAGE. The HC bands (HEK Trastuzumab, Herceptin and all mutants except Q160N) and the LC band (Q160N) were excised from the gel and diced. The proteins in the gel were reduced by incubation with 10 mM DTT at 56 °C for 45 min and then alkylated with 55 mM iodoacetamide (IAA) in 100 mM NH_4_HCO_3_ at 25 °C for 30 min. The alkylated proteins were digested with trypsin (1 µg per 50 µg of protein) in 50 mM NH_4_HCO_3_ (pH 6.8) at 37 °C overnight. The tryptic peptides were subsequently treated with chymotrypsin in 100 mM Tris-HCl and 10 mM CaCl_2_ (pH 7.8) at 25 °C overnight. C18 ZipTips were used for desalting the tryptic/chymotryptic peptides, using 80% (*v*/*v*) acetonitrile in 0.1% TFA for elution. Solvent was removed by vacuum centrifugation and the peptides were dissolved in 0.1% formic acid for LC–MS/MS analysis.

#### 2.3.2. Free Glycan Analysis

The glycans were released from the antibody and analyzed through tandem mass spectrometry as described previously [27]. Briefly, antibody aliquots were reduced and alkylated using 1 M DTT and 500 mM IAA, respectively. Subsequently, 10 µg of protein was blotted on a PVDF membrane previously wetted with ethanol and left to dry overnight. The membrane was washed with methanol, followed by a wash with water to remove salts. The membrane was stained briefly with Direct Blue 71 solution (1 part 0.1% Direct Blue 71 stain (*w*/*v*) in MilliQ water and 12 parts wash solution (40% ethanol (*v*/*v*), 10% acetic acid (*v*/*v*)) to visualize the spots. The spots were briefly de-stained and washed with water. The spots were cut from the membrane and transferred to 96-well plates and blocked with 1% (*w*/*v*) polyvinylpyrrolidone 40 (PVP40). The spots were washed three times with water and placed in new wells containing water. PNGase F was subsequently added to the wells (1 unit per 5 ug of protein) and the plate was incubated overnight at 37 °C. The resulting solution containing the released glycans was collected and transferred to a low-protein binding tube. A volume of 10 µL of 100 mM ammonium acetate pH 5 was added followed by incubation for 1 h at 25 °C to remove glycosylamines from the reducing end. The samples were dried by vacuum centrifugation followed by reduction with 1 M NaBH_4_ in 50 mM KOH for 3 h at 50 °C. The reaction was neutralized with 1 µL glacial acetic acid.

An AG 50W X8 cation exchange resin loaded onto a ZipTip C18 tip for packing was used for desalting the reduced glycans. Prior to sample loading, the columns were washed three times with 50 µL of 1 M HCl followed by three washes with 50 µL of methanol. The columns were then transferred to new collection tubes and washed three times with 50 µL of water. The samples were loaded onto the column and eluted with water. The eluted glycans were dried with a SpeedVac concentrator and redissolved in 100 µL methanol to remove residual methyl borate and dried once more. Methanol redissolution was performed three times.

Finally, a carbon cleanup step was performed with a carbon solid-phase extraction (SPE) slurry packed into a TopTip. The carbon SPE columns were initially washed three times with 50 µL of acetonitrile in 0.1% (*v*/*v*) TFA. The columns were then washed three times with 50 µL of 0.1% (*v*/*v*) TFA in water. The desalted reduced glycans were dissolved in 0.1% (*v*/*v*) TFA in water and loaded onto the column. The columns were washed three times with 50 µL of 0.1% (*v*/*v*) TFA in water and the glycans were eluted with 50% (*v*/*v*) acetonitrile in 0.1% (*v*/*v*) TFA. The glycans were dried in a SpeedVac concentrator and dissolved in 10 mM NH_4_HCO_3_ for LC–MS/MS analysis.

### 2.4. Mass Spectrometry

Glycopeptide analysis was performed using an in-house packed 20 cm × 75 µm Reprosil-Pur C18AQ (3 µm, 120 A) coupled to an Orbitrap Fusion MS (Thermo Scientific) for LC–MS/MS. HPLC solvent A was 0.1% (*v*/*v*) formic acid and solvent B was 80% (*v*/*v*) acetonitrile in 0.1% formic acid. The peptides were separated running a 90 min 0−40% solvent B gradient at 300 nL/min at 60 °C. The Orbitrap Fusion mass spectrometer was used in positive mode, with source voltage = 2.3 kV, S lens RF level = 68%, and a capillary temperature of 275 °C. Initial MS scan was acquired from 350 to 2000 m/z at a resolution of 60,000 at 400 m/z (MS AGC = 6 × 105). Following MS1, data-dependent higher-energy collisional dissociation (HCD) was used at top speed. HCD parameters were set up as: activation time = 0.1 ms, maximum injection time = 200 ms, dynamic exclusion = enabled with repeat count 1, resolution = 30,000, normalized energy = 40, exclusion duration = 20 s, default charge state = 2 and MSn AGC = 2.0 × 105. The two most intense precursors were re-isolated and subjected to collision induced fragmentation (CID). CID parameters were: resolution = 30,000 in orbitrap, activation time = 10 ms, dynamic exclusion = enabled with repeat count 1, normalized energy = 35, exclusion duration = 20 s, default charge state = 2, MSn AGC = 5.0 × 104.

MS analysis of the free glycans was performed on a VelosPro (Thermo) mass spectrometer with an Agilent 1260 HPLC using a Hypercarb porous graphitised carbon capillary column (3 µm particle size, 100 mm × 180 µm, pore size 250 Å, Thermo). The glycans were separated using a 60 min 5–45% (*v*/*v*) solvent B (80% (*v*/*v*) acetonitrile in 10 mM NH4HCO3) gradient on a flow rate of 2 μL/min. The column was washed with 100% solvent B and equilibrated with solvent A (10 mM ammonium bicarbonate) after each run. ESI–MS was performed in negative ion mode at a resolution of 30,000 with a mass range of m/z 500–2000. Transfer capillary temperature was at 275 °C and the capillary voltage was 3 kV. MS2 of the top 9 most intense ions was performed using collusion induced dissociation (CID) at a normalized collision energy of 35 in the ion trap with an activation time of 10 ms.

### 2.5. Data Analysis

Analysis of the peptide and glycopeptide mass spectrometry data was performed using Byonic^TM^ 3.0 (Protein Metrics Inc., Cupertino, CA, USA) software. Peptide spectrum matches (PSM) were obtained through a search against the FASTA files of the control Trastuzumab and its variants. CID spectra annotation was automated using Byonic and the spectral matches were inspected manually. The Byonic search was conducted with a semi-specific digestion specificity allowing two missed cleavages. The fragment mass tolerance was 0.05 Dalton (Da). A maximum of 2 common modifications (asparagine and glutamine deamidation +0.984016 Da, cysteine carbamidomethylation +57.021464) and 1 rare (methionine oxidation +15.994915) modifications were enabled.

Free glycans were analysed manually using Thermo Xcalibur Qual Browser version 3.0.63. The theoretical monosaccharide compositions were determined using the monoisotopic masses of the detected ions using GlycoMod (http://web.expasy.org/glycomod/ accessed on 1 July 2019) with mass tolerance of ±0.5 Da. The glycan structures were manually assigned using PGC retention time and MS2 diagnostic fragment ion information. The percentage relative quantitation of each glycan structure per Tmab variant was calculated using the extracted ion chromatogram (EIC) areas upon peak smoothing using Gaussian Algorithm (15 points). The area values for the glycan structures were summed and normalized to 100% and each glycan peak was expressed as percentage of the total.

### 2.6. Binding Affinity to HER2 and Fc Receptors

The control Trastuzumab references (HEK Trastuzumab and Herceptin) and the hyperglycosylated mutants were tested for their capacity to bind their molecular target (HER2) and Fc receptors (FcγR1A, FcγR2B, and FcγR3A). A Biacore T200 instrument (GE Healthcare, Parramatta, Australia) was used to obtain the binding kinetic constants through surface-plasmon resonance (SPR) using single-cycle kinetic analysis as reported in our previous paper [26].

For HER2 binding experiments, a CM5 sensor chip was functionalized with an anti-HIS antibody through amine coupling chemistry. The HIS-tagged HER2 receptor (4 nM) was bound to the anti-HIS at 5 µL/min flow rate for 5 min. The Trastuzumab variants were assayed in single-cycle kinetic titrations in 2-fold serial dilutions spanning 0.5–8 nM using HBS-T running buffer (10 mM HEPES, 150 mM NaCl, 0.05% (*v*/*v*) Tween 20, pH 7.4). Analyte runs were performed at 20 µL/min for 2 min with 60 min dissociation times. All experiments were conducted in duplicate.

HIS-tagged Fc receptors were bound to the anti-HIS antibody on the CM5 sensor following the same procedure as for HER2. The analyte runs were performed as described above for HER2, with the exception of a 30 min dissociation time and that analyte concentrations ranged from 2.5 to 40 nM for FcγR1A, and from 25 to 400 nM for FcγR2B and FcγR3A.

The sensorgrams were fitted to a bivalent binding model as the analyte (antibody) has two binding sites. Binding constants (*K_D_*) were obtained for all samples, and where kinetics was observed, the on rate and off-rate (*K_a_* and *K_d_*) were also determined.

### 2.7. Melting Temperature (T_m_) and Onset Aggregation Temperature (T_agg_) of Proteins

Using an UNcle^TM^ system (Unchained Labs, Pleasanton, CA, USA), the *T_m_* and *T_agg_* of proteins were simultaneously probed by intrinsic tryptophan fluorescence and static light scattering (SLS), respectively and as described previously [26]. Briefly, using a laser excitation at 266 nm, intrinsic tryptophan fluorescence and SLS were simultaneously recorded during a linear temperature scan between 15 and 95 °C with a scan rate of 0.5 °C/min, and with no holding time in order to maximise the frequency of detection. Each sample was measured in triplicates in the UNcle^TM^ UNI sample holder that contains 16 quartz cells. Data analysis was performed using the UNcle^TM^ Analysis software and the barycentric mean (BCM) was used to plot the *T_m_* curves over 300–450 nm range, which is defined by the Equation:

(1) $$\lambda_{\mathrm{BCM}}=\left(\sum_{\lambda }\lambda I\left(\lambda \right)\right)/\left(\sum_{\lambda }I\left(\lambda \right)\right)$$

Here, λ is wavelength in nanometers, I is fluorescence intensity in that particular wavelength. This process provides an averaged peak wavelength ($\lambda_{\mathrm{BCM}}$) within the given wavelength range.

## 3. Results

### 3.1. Engineered Glycosylation Sites

Table 1 lists the surface-exposed amino acid residues that were mutated herein in Trastuzumab with the respective proposed mechanism to increase physical stability. One residue (L115) lies on the variable region of the heavy chain (V_H_), five (A121, L177, Q178, L182 and T198) on the constant heavy (C_H_1) and one on the constant region of the light kappa chain (C_k_). The amino acid substitutions L115N, Q160N, Q178N and L182N were derived from previous studies by Courtois et al. that identified sites on the tertiary structure of the Fab region of IgG1 antibodies where the incorporation of a glycan could shield hydrophobic surface-exposed regions. These aggregation-prone regions (APRs), or aggregation hotspots, were mapped with the Spatial Aggregation Propensity (SAP) technology, wherein individual amino acids are assigned an aggregation propensity value based on side chain hydrophobicity, solvent-accessible surface area (SASA), the hydrophobic contributions of adjacent amino acids within a given radius, and molecular dynamic (MD) simulations [25]. For more details on this technique see [22].

Aside from T198, all other the aforementioned substitutions were engineered to shield the APR generated by the high SAP residue L177. Mutations L115N and Q160N have previously demonstrated to improve resistance to aggregation through accelerated stability studies in bevacizumab—a therapeutic monoclonal antibody with a pronounced tendency to aggregate through self-association [25]. Q178N and L182N were identified in the same study as positions in the vicinity of L177N that can be mutated to generate an *N*-glycosylation sequon (Asn-X-Ser/Thr) through a single-amino acid substitution; however, these were excluded from experimental tests in bevacizumab due to Q178 having reduced SASA and L182 not being oriented on the same face as the APR. In this work, L177 was also mutated to substitute the high SAP residue with an asparagine within a consensus motif for glycan addition. The latter mutation was recently tested in an adalimumab Fab expressed in *Pichia pastoris*, conferring improved resistance to aggregation and proteinase K digestion relative to wild-type adalimumab Fab [28].

A121N, Q178N and T198N were identified by Pepinsky et al. [23] as glycosite additions that could prevent self-association through steric hindrance in an anti-LINGO antibody with reduced solubility. In this case, all three modifications granted a dramatic improvement in solubility.

### 3.2. Glycan Occupancy Prediction

**Figure 1:**
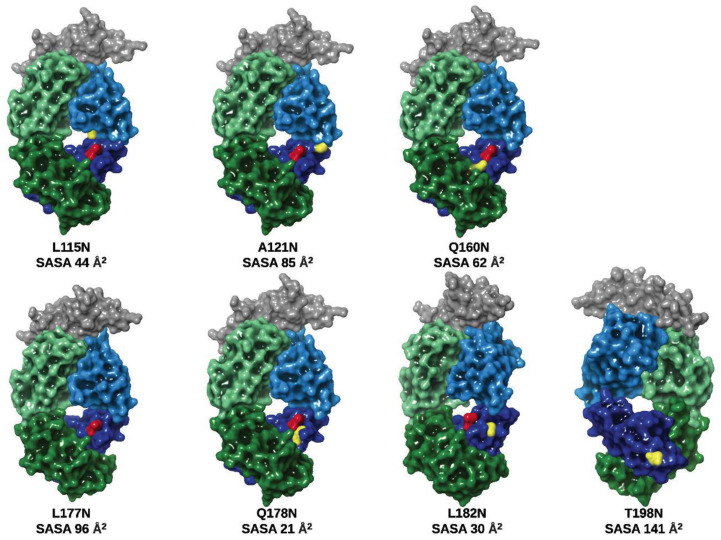
Location of the mutated amino acid residues for glycosite addition. Crystal structure of Table 2. extracellular domain complexed with the Fab of Trastuzumab (1N8Z) visualized in Maestro highlighting the solvent-exposed surface area of the mutated amino acid positions. The mutated amino acids are highlighted in yellow. The high SAP L177 is displayed in red. VH and CH1 domains are shown in light blue and dark blue, respectively. VL and CL domains are shown in light green and dark green, respectively. A trimmed section of the HER2 ECD is displayed in dark grey.

To assess the validity of employing existing algorithms for the prediction of glycan attachment, the primary sequence of the Trastuzumab variants was subjected to an in silico analysis using two available *N*-glycosylation prediction servers. Both servers, NetNGlyc 1.0 and NGlycPred, assign a 0–1 score, wherein values greater than 0.5 indicate a predicted glycosylated asparagine residue. Figure 1 depicts the location and surface exposure of the amino acid substitutions and the high SAP L177. Table 2 reports the predicted potential for glycan occupancy obtained with the NetNGlyc 1.0 server, which relies on adjacent primary sequence to assign a probability for N-glycan transfer during protein translation. This server has been employed successfully in various studies to predict glycosylation in identified Asn-X-Ser/Thr motifs within primary amino acid sequences [29,30,31]. NetNGlyc predicted all residues, except Q178N, to undergo glycosylation. Further, an additional algorithm (NGlycPred) that incorporates structural features (e.g., adjacent secondary structure, surface accessibility, local contact order) into the analysis was utilized for prediction based on fundamentally distinct inputs [32]. NGlycPred assigned a negative value (<0.5) to L115N and Q178N.

### 3.3. Glycan Attachment Confirmation

All listed mutations were implemented and expressed in HEK-293F human embryonic kidney cells. The attached glycans were subsequently released by PNGase F digestion and analysed by capillary/nanoLC–ESI–MS/MS to confirm glycan attachment and obtain structural features of the oligosaccharides. Glycan attachment on the mutated sites was analysed through electrophoretic mobility shifts in SDS-PAGE and MS/MS glycan profile analysis. A size shift for the heavy-chain band was observed for the glycomutants on SDS-PAGE (Figure S1). The heavy-chain (HC) band (~50 kDa) mobilities of the L115N, A121N, L177N, Q178N, L182N, and T198N mutants were retarded relative to that of HEK Trastuzumab, commercial Herceptin (Herc) and Q160N (mutated on the light chain), indicating higher levels of glycosylation on these HC mutants.

Interestingly, no peptide spectral matches were found for glycopeptides containing the L177N, Q178N and L182N residues. Conversely, peptides containing the non-mutated L177, Q178 and L182 amino acids were detected in HEK Trastuzumab and Tmab variants with mutations outside the region (Figures S32–S34). Assuming no missed cleavages, the tryptic/chymotryptic peptide containing L177N, Q178N or L182N has a mass of 2206.11 Da (Table 3). It is likely that glycan attachment on the side chain hinders enzymatic digestion, thus producing peptides with missed cleavages and m/z values too large for detection—glycan attachment increases the m/z further. Large glycopeptides can be missed in the workflow utilized for this analysis during peptide extraction following SDS-PAGE, as the large size could prevent them from extraction due to gel shrinkage caused by the organic solvents employed [27]. Similarly, glycan attachment in the vicinity of the cleavage sites has been shown to prevent enzymatic activity of the serine protease proteinase K in the L177N mutant in adalimumab Fab [28]. Still, successful glycosylation of L177N, Q178N, and L182N was confirmed by mobility shifts in SDS-PAGE and increased heterogeneity observed in the released glycan profile compared to HEK Trastuzumab as discussed in the next section.

**Figure 2:**
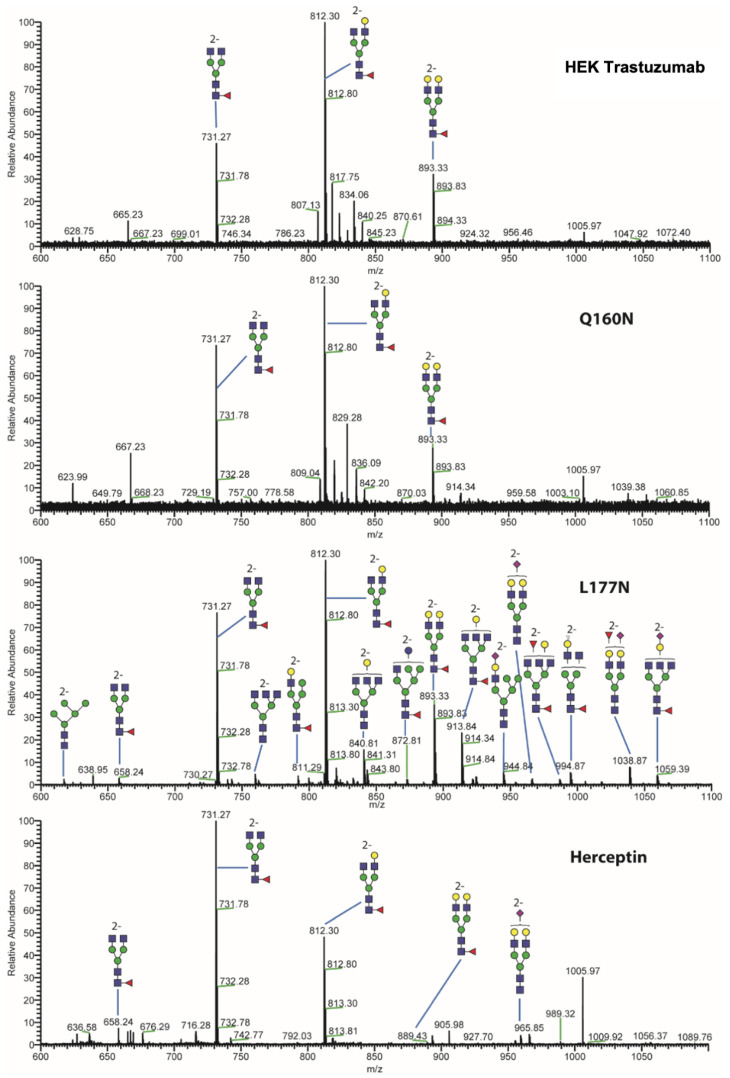
MS spectra of *N*-glycans released from HEK Trastuzumab, Q160N, L177N and Herceptin contrasting the heterogeneity found on the Fab glycoengineered Trastuzumab.

Despite the positive prediction from the algorithms (NGlycPred score of 0.834 (Q160N), Table 2), the light-chain (LC) band (~25 kDa) of Q160N showed no apparent mobility shift compared to HEK Trastuzumab LC region, suggesting a lack of glycosylation on the mutated asparagine residue. Although the glycan profiling of the Q160N showed very similar structures to HEK Trastuzumab, the glycan release was performed on the whole protein but not on the light chain only and therefore provide an overall glycoprofile of the protein (Figure 2, Table 4). While the visual similarity of the light-chain gel mobilities of HEK Trastuzumab and Q160N and the information of the overall glycan profile of Q160N do not entirely exclude the possibility of glycan occupancy on the 160N, the MS analysis of the Q160N light-chain peptide clearly showed the presence of a non-glycosylated LC region. To confirm the lack of glycan attachment, the Q160N mutant was subjected to trypsin and chymotrypsin digestion to generate the corresponding peptides for nanoLC–ESI–MS/MS analysis. Spectral matches were found for non-glycosylated peptides encompassing the Q160N residue, thus confirming successful Q to N conversion and reaffirming the lack of glycan attachment (Figure S35). This apparent lack of glycan attachment was also observed in the corresponding Q160N mutant when expressed in adalimumab (Q161N) [26].

### 3.4. Glycan Profile Analysis

**Figure 3:**
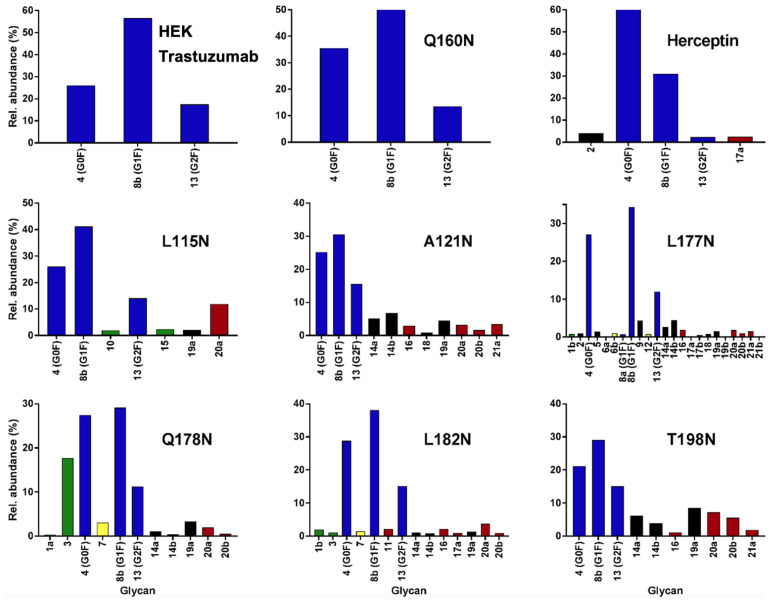
Relative abundances of the glycan found on each specific mutant. Highlighted in blue are G0F, G1F and G2F which are also present on HEK Trastuzumab. High mannose glycans are displayed in green, hybrid glycans in yellow and sialic acid containing glycans in red. The percentage relative quantitation of each glycan structure per Tmab variant was calculated using the extracted ion chromatogram (EIC) areas upon peak smoothing using Gaussian Algorithm (15 points). The area values for the glycan structures were summed and normalized to 100% and each glycan peak was expressed as percentage of the total.

To analyze the global glycan profile of the Trastuzumab variants, the proteins were immobilized on PVDF membranes and treated with PNGase F to release glycans attached to both the conserved N297 and the added glycosylation site. The released oligosaccharides were run on capillary LC–ESI–MS/MS, wherein a PGC column was used for separation of distinct glycoforms and MS/MS analysis (CID fragmentation) was performed to elucidate glycan structure. Figure 2 shows the annotated full MS spectra of HEK Trastuzumab, Herceptin, Q160N, and L177N displaying the identified glycan structures found on each variant. The global glycan profile reflects contrasting differences in glycoform heterogeneity and the relative abundance of the glycans found on the glycomutants. MS1 and MS2 (where possible) spectra of all the glycomutants can be found in Supplementary Materials. The released glycan profiles displayed in Figure 2 and Figures S2–S10 come from both the conserved Fc sequon and the added glycosylation site. Table 4 lists the glycan structures found on all Tmab variants through this MS methodology; and Figure 3 reports the glycan relative abundances on specific Tmab variants determined by quantifying the area under the curve (AUC) of the extracted ion chromatograms.

Glycan microheterogeneity at the conserved N297 in HEK Trastuzumab is similar to that reported in previous analysis of commercial Herceptin and other monoclonal therapeutic antibodies, where complex G0F, G1F and G2F glycoforms (Figure 3 and Table 4) are predominant [33,34]. Minor species (i.e., Man5, G0 and G2S1) were detected in commercial Herceptin but not in HEK Trastuzumab (Figure 2). An important difference between commercial Herceptin and HEK Trastuzumab lies in the relative abundance of the three main glycoforms (G0F, G1F and G2F), where the originator contained the pattern G0F > G1F > G2F (60%, 31%, and 2%) and HEK Trastuzumab possessed G1F > G0F > G2F (26%, 57%, and 17%) (Figure 3). Glycosylation differences are expected between these two samples, as the originator is produced in CHO cells and our in-house Tmab in HEK-293F. Moreover, sialic acid-containing species (17a Table 4) were present in the originator and not detected in HEK Trastuzumab. This observation is consistent with previous studies reporting higher sialylation of *N*-glycans in recombinant proteins expressed in CHO when compared to HEK cells [35].

**Figure 4:**
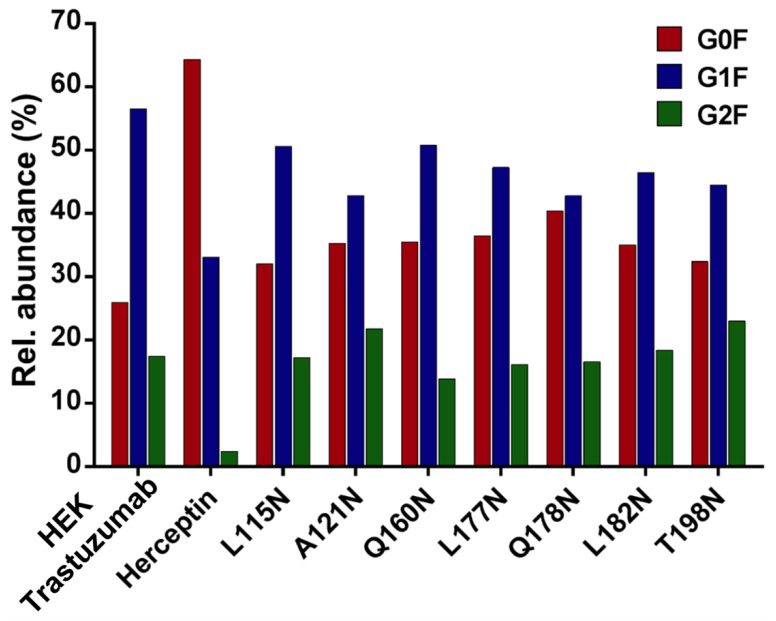
Relative abundances of the main glycoforms G0F, G1F and G2F found on all Trastuzumab variants. The percentage relative quantitation of each glycan structure per Tmab variant was calculated using the extracted ion chromatogram (EIC) areas upon peak smoothing using Gaussian Algorithm (15 points). The area values for these particular glycan structures were summed and normalized to 100%, without accounting for other less abundant glycoforms. Each glycan peak was expressed as percentage of the total for the main glycoforms.

The G1F > G0F > G2F distribution observed in HEK Trastuzumab was conserved in all the glycomutants (Figure 3), although the abundance of G1F seems to decrease in comparison to that observed for HEK Trastuzumab. Figure 4 shows the relative abundances considering only the main complex glycoforms found on the Fc domain (G0F, G1F, and G2F). The decrease in G1F and concomitant increase in G0F was more pronounced in the Q178N mutant where G1F (40.47%) was only slightly less abundant than G0F (42.98%) (Figure 4).

Most notably, a much wider heterogeneity of glycan structures was detected on most Tmab variants relative to control Trastuzumab (HEK Trastuzumab and Herceptin) (Figure 3 and Figure 4). The fact that G0F, G1F, and G2F are still the most abundant structures in all samples indicates that the increased variety of glycans likely come from the Fab region. The wider array of structures includes high mannose, hybrid, and complex biantennary, triantennary and tetrantennary glycans with varied degrees of galactosylation and sialylation (Table 4). Variants L177N and L182N displayed the most varied sets of glycoforms with 16 and 12 different structures detected, respectively. Importantly, sialic acid-containing glycans were particularly abundant in L115N, L182N, and T198N. Q178N had a significant presence of high mannose glycans.

### 3.5. Binding Affinity to HER2 and Fc Receptors

To assess potential alterations in biological activity caused by the structural modifications of glycan addition, we tested the binding affinity of the Tmab variants on biologically relevant receptors in vitro. Surface-plasmon resonance (SPR) single-cycle kinetic assays were performed to determine binding constants of the Tmab variants to their molecular target HER2, and to Fc receptors FcγR1A, FcγR2B, and FcγR3A (Table 5 and Table 6).

SPR-derived binding kinetic parameters of the mutants on immobilized HER2 displayed a reduction in binding affinity (*K_D_*) compared to control Trastuzumab (Table 5). *K_D_* values for the Tmab variants exhibited a 3–30-fold decrease in binding affinity, with affinities ranging from 110 to 1160 pM compared to 30 pM for HEK Trastuzumab. The reduction in binding affinity to HER2 was due to the faster off-rates observed; generally between 2- and 10-fold faster (Table 5). Despite this, all mutants bar L115N still exhibited sub-nM binding affinities and while several-fold weaker, variants L182N and Q160N exhibit binding in a comparable order of magnitude, with only slightly faster off-rates.

Binding affinity to captured FcγR1A for all mutants was comparable to both HEK Trastuzumab and Herceptin (10 nM and 5 nM, respectively), exhibiting *K_D_* values between 3 and 8 nM. The kinetic parameters (*K_a_* and *K_d_*) were similarly comparable across all mutants (Table 5).

SPR assays indicated both HEK Trastuzumab and Herceptin bind to FcγR2B; however, the kinetic parameters were outside the range that can be measured by the instrument—note the square shape of the sensorgrams in Figure S38, indicative of very fast on and off rates. Due to this, fits to the responses obtained at equilibrium were instead used to determine *K_D_* values (Table 6). Where a response was able to be measured, generally affinities of the mutants were within the same order of magnitude; with binding between 2-fold stronger and up to 4-fold weaker.

Similarly, HEK Trastuzumab and Herceptin were shown to bind to FcγR3A; however, for HEK Trastuzumab and some of the mutants, the kinetic parameters were outside the range that can be measured by the instrument. To obtain consistent measurements across the data set, fits to the responses obtained at equilibrium were again used to determine *K_D_* values (Table 6). All the measured mutants yielded similar binding affinities; with values obtained between 220 and 600 nM. These affinities were comparable to those obtained from HEK Trastuzumab and Herceptin of 270 nM and 430 nM, respectively.

### 3.6. T_m_ and T_agg_

Melting (*T_m_*) and aggregation onset temperatures (*T_agg_*) of proteins provide structural stability and aggregation propensity information. These parameters varied greatly for mutated proteins as shown in Table 7. Full monoclonal antibodies generally have at least two melting temperatures (*T_m_*1 and *T_m_*2) due to Fc and Fab fragments unfolding independently. L115N, L177N and L182N had increased *T_m_*1 (73, 71, 73 °C, respectively) compared to Herceptin^®^ (68 °C) and Tmab (68 °C) indicating that they have higher structural stabilities than Herceptin and Tmab for this domain. However, only L115N has a small increase in *T_m_*2 (84 °C) compared to Herceptin^®^ and Tmab (82 and 83 °C, respectively).

All variant and Tmab had much higher *T_agg_* than Herceptin^®^ (81 °C). However, only L115N and T198N had approximately 6 and 3 °C, respectively, higher *T_agg_* than Tmab (85 °C).

## 4. Discussion

The insertion of novel *N*-glycosylation sites on the primary structure of IgG1 molecules is a promising approach to enhance the physicochemical properties of therapeutic proteins, having previously demonstrated important enhancements in solubility and physical stability in bevacizumab, adalimumab, and the Li33 anti-LINGO antibody. However, implementation of this approach in clinical development could be challenging due to the concomitant increase in structural heterogeneity. This is especially relevant when considering mAbs whose therapeutic efficacy relies, at least partially, on eliciting effector functions; given that the technique entails the incorporation of a relatively large molecule that should not alter detrimentally the binding affinity towards the various receptors involved in its biological activity (i.e., cognate antigen, Fc receptors, C1q). Further considerations include potential modifications in immunogenicity and pharmacokinetic profiles. Moreover, elucidation of the underlying mechanisms that drive improvements in stability demands thorough structural analysis of the glycan structures attached to the engineered site. In light of this, we inserted *N*-glycosylation motifs in Trastuzumab in several amino acid positions previously identified by others on conserved regions of the IgG1 molecule and performed a detailed structural analysis of the ensuing glycan profile.

### 4.1. Preliminary Prediction and Confirmation of Glycan Attachment

*N*-glycosylation is a complex biochemical process, for which the requirements for glycan attachment are not yet fully understood. Three key factors determining glycan occupancy have been identified: (1) location of the asparagine residue within the consensus motif Asn-X-Ser/Thr, (2) location of the N residue in the ER lumen during translation, and (3) the adjacent secondary structure of the protein must enable glycan transfer. Based on these, numerous servers have been developed to map *N*-glycosylation sequons and predict the probability of glycan attachment and have been instrumental in the study of the glycosylation profile of various proteomes. We thus employed two such servers to assess the validity of their prediction values applied to this antibody engineering strategy. Previous studies have not pursued potential APR shielding regions experimentally due to low glycan transfer probability. Specifically, residue Q178 was deemed ineligible by Courtois et al. based on low solvent-accessible surface area obtained from MD simulations. Our preliminary evaluation of glycan attachment probability on Trastuzumab shed similar results (Table 2) to those obtained by Courtois et al. on their panel of glycosylation sites identified through SAP, wherein Q178N had negative predictions by both NetNGlyc and NGlycPred, and SASA was amongst the lowest calculated (20.5 Å^2^). Importantly, L115N also obtained a negative prediction value from NGlycPred. It was thus remarkable that our experimental data contradicted the negative prediction for residues L115N and Q178N. The Q178N mutant had previously been implemented by Pepinsky et al. in the anti-LINGO IgG1 antibody with important improvements in solubility, although no compelling data to confirm glycan attachment was reported. Moreover, our data strongly suggest that Q160N does not undergo glycan attachment, despite the high SASA and prediction values calculated for the residue. This was equally observed in the equivalent mutation in adalimumab [26]. These results emphasize the importance of confirming glycan attachment and performing structural analysis of the resulting structural modifications, to better interpret the results derived from aggregation experiments. Furthermore, they reveal a conspicuous need to further study the factors that determine successful glycan transfer to refine this antibody engineering approach.

### 4.2. Glycan Profile Analysis

Compared to plasma proteins and other recombinant therapeutic proteins, monoclonal antibodies display limited heterogeneity in their Fc *N*-glycan profile. This observation is ascribed to the decreased surface exposure of Asn N297 and the attached glycan as it engages in several interactions with the protein backbone and residue side chains at the inner face of the domain [35,36]. Although most plasma IgG molecules possess only the conserved *N*-glycosylation sequon in the Fc region, approximately 15–25% also express additional glycosylation sites on the variable domain of the Fab region [37,38]. Glycan profiling of Fab-glycosylated serum IgGs has revealed contrasting structural characteristics between Fc-derived and Fab-derived glycans, most notably higher overall heterogeneity and sialylation [39,40]. All mutations evaluated in this study are located in the Fab region, although only L115N lies on a variable domain—in close proximity to the V_H_-C_H_1 interface. The global glycan profile acquired through capillary/nanoLC–ESI–MS/MS demonstrates that increased structural heterogeneity is similarly obtained when engineered glycosylation sites are incorporated through mutagenesis in the Fab region of a recombinant mAb.

There was no evident association between the degree of heterogeneity or apparent glycan maturation and the solvent-accessible surface area calculated. For instance, low-SASA mutant L115N possessed a relatively high abundance of sialylated glycans, indicative of higher glycan processing. Remarkably, high mannose glycans (HexNAc(2)Man(8) and HexNAc(2)Man(9)) were also detected in L115N. Similarly, residue Q178N displayed high glycoform heterogeneity despite having the lowest SASA amongst the mutants, including sialylated and triantennary glycans. As the en bloc transfer of the dolichol oligosaccharide precursor occurs on nascent proteins undergoing translation, low surface exposure calculated from the non-mutated mAb structure might not preclude glycan attachment and the resulting orientation of the glycan could be surface-exposed for enzyme accessibility.

Overall, the degree of sialylation was markedly higher in most engineered mutants relative to both control references, i.e., HEK Trastuzumab and Herceptin. Similar findings have consistently been reported for both polyclonal and recombinant monoclonal IgG proteins containing naturally-occurring *N*-glycosylation sequons in the Fab region [41]. The higher abundance of sialic acid glycans in Fab glycosites has been attributed to increased accessibility to enzymes involved in glycan maturation relative to the Fc glycan. In this case, however, we also found various high mannose and hybrid glycans not detected in HEK Trastuzumab. In the seminal exploration of SAP reduction by Courtois et al., a G0 glycan was selected for molecular dynamic simulations of the Fab region to shed light into the factors driving resistance to aggregation upon glycosite insertion [25]. Here, it was posited that the added glycans on some of the mutated sites produce limited APR shielding and hence the improvements in physical stability could stem from steric hindrance due to the large hydrodynamic size and hydrophilic character of the added glycans. The diversity of glycoforms reported here indicates that this structural heterogeneity must be accounted for when performing these analyses, especially in account of the important presence of negatively charged sialic acid residues on some of the mutants.

HEK-293F cells were chosen for transient expression of the Tmab variants due to high transient transfection efficiency and yield relative to CHO cells. Comparison of the glycan profile of CHO-produced Herceptin with HEK-derived Tmab shows that galactosylation was more abundant in HEK-293F expression. This observation was also consistent across the mutant panel when considering uniquely Fc glycans. Galactosylation of the conserved Fc glycan is not considered to affect significantly mAb binding to FcγR receptors, yet there is consistent evidence that the presence of terminal galactose promotes activation of the classical complement route [42]. Hence, the higher content of G1F and G2F glycoforms could lead to potency advantages in mAbs with significant modulation of complement activity.

### 4.3. Alterations in HER2 and Fc Receptor Affinity

An important consideration in this engineering approach must be the conservation of biological activity. The proximity of the mutations to the CDRs and to regions involved in Fc receptor binding warrants examination of alterations in receptor binding affinity. Thus, we evaluated the binding kinetics of the panel of mutants to their molecular target (HER2) and to Fc receptors involved in effector function.

*K_D_* values derived for control Tmab binding to HER2 are in the same order of magnitude with those reported in previous literature, spanning 19 pM–6 nM [43,44,45]. The kinetic constants obtained indicate that HER2 binding affinity is diminished for all mutants, reflected by 3–30-fold reductions in the binding affinity constant (*K_D_*). Other than L115N, all *K_D_* values were subnanomolar—demonstrating that strong binding to HER2 is retained despite glycosylation clearly affecting binding affinity.

Based on our calculations, L115N was by far the weakest binder, along with 121 and 198. T198N is located farthest from the HER2 binding region. This reduction in binding affinity could be due to conformational changes affecting the antigen binding region.

Overall, the mutant library exhibited minor modifications in Fc receptor binding affinity. Binding to FcγR1A and FcγR3A was comparable to HEK Trastuzumab for all Tmab variants. Equilibrium fits for FcγR2B indicated comparable or weaker binding to some mutants; however, others either exhibited low responses (Q178N, T198N), or no observable binding (L182N) over the binding range assayed.

The affinity screening to biologically relevant receptors performed in this work serves as a proof-of-principle that *N*-glycosylation sites can be incorporated in the Fab region of therapeutic monoclonal antibodies without major modifications in antigen and Fc receptor binding. Still, evaluation of ADCC, CDC and inhibition of cell proliferation is still required to ascertain potential variations in biological activity.

### 4.4. Structural Stability and Aggregation Propensity

A crucial aspect in protein engineering approach is the structural stability and aggregation propensity of proteins. This is especially important if we need to move from intravenous infusions to much desired pre-filled syringe formulations, which require high protein concentrations. In such formulations, structural stability as well as aggregation tendency play a critical role, in particular during long-term storage conditions. Based on our accelerated experiment where we determined *T_m_* and *T_agg_* of mutants, all mutants behaved similar to or better than Tmab, mainly in aggregation tests. Two mutants (L115N and T198N) displayed much enhanced *T_agg_* compared to other proteins. This is perhaps not surprising since glycosylation is known to generally stabilize proteins. Having bulky glycans close to the aggregation-prone regions on protein surface would prevent protein-protein interactions that would lead to aggregation.

## 5. Conclusions

The rational addition of *N*-glycosylation sequons represents a promising strategy to improve the physical stability of therapeutic antibodies, as is highlighted by the important enhancements in physical stability obtained in previous studies with bevacizumab, adalimumab Fab, and the Li33 anti-LINGO antibody. Moreover, we have previously performed the panel of mutations reported here in full-size adalimumab and demonstrated enhancements in thermodynamic stability through this approach [26]. However, our data establish that for this strategy to move forward in the clinical development pathway, there is an evident need to control or minimize the structural heterogeneity that results from glycan addition. The latter is also required to obtain insight into the conferred physicochemical properties responsible for the improvements in stability. Native Fc glycosylation, albeit less diverse than most glycoproteins, already constitutes a challenge concerning the assessment of critical quality attributes. This, however, could be overcome with glyco-engineering strategies to curtail glycoform heterogeneity. Technologies to reduce glycan diversity in recombinant expression are being pursued in the context of refining the production of biopharmaceuticals. Prime examples of these include engineered *Pichia pastoris* (GlycoSwitch) and human (GlycoDelete) cell lines. We foresee great value in combining these two glyco-engineering strategies towards the development of therapeutic antibodies with enhanced physicochemical and biological properties. Albeit introducing heterogeneity, molecular engineering approaches such as this one offer a great deal of flexibility and control over preparation of new-generation therapies including biobetters. This glycoengineering strategy can also be employed to enhance the biophysical stability of other protein-based therapies with short half-life and low formulation stabilities.

## Figures and Tables

**Table 1:** Surface-exposed amino acid (aa) residues identified in Tmab Fab region for glycosylation sequon addition.

aa Substitution (Position)	Region	Proposed Function
L115N	V_H_	Mask APR by introducing glycosylation site [26]
A121N	C_H_1	Improve solubility by introducing glycosylation site [25]
L177N		Mutate aa with high spatial-aggregation propensity and introduce glycosylation site [26]
Q178N		Sterically hinder self-association by introducing glycosylation site and increase solubility [25,26]
L182N		Mask APR by introducing glycosylation site [26]
T198N		Improve solubility and sterically hinder self-association by introducing glycosylation site [25]
Q160N	C_k_	Mask APR by introducing glycosylation site [26]

**Table 2:** Estimated glycosylation potential and solvent-accessible surface area (SASA) of the targeted *N*-glycan sequons.

aa Substitution (Position)	Region	Sequon	NetNGlyc	NGlycPred	SASA (Å^2^)
Q160N	C_K_	160 NESV	0.647	0.834	62.478
L115N	V_H_	115 NVTV	0.679	0.462	43.709
A121N	C_H_1	121 NSTK	0.547	0.686	85.338
L177N	C_H_1	177 NQSS	0.537	1.000	96.039
Q178N		178 NSSG	0	0.157	20.5
L182N		182 NYSL	0.568	0.999	30.123
T198N		198 NQTY	0.592	1.000	141.149

**Table 3:** Trypsin/chymotrypsin peptides spanning the L177N, Q178N, L182N and T198N mutations allowing one missed cleavage (MC) site. Mutated position are in bold.

Mass (Da)	Position	MC	Sequence
4000.01	162–201	1	NSGALTSGVHTFPAV**LQ**SSG**L**YSLSSVVTVPSSSLGTQTY
3248.63	153–183	1	FPEPVTVSWNSGALTSGVHTFPAV**LQ**SSG**L**Y
3148.59	184–213	1	SLSSVVTVPSSSLG**T**QTYIC NVNHKPSNTK
2206.11	162–183	0	NSGALTSGVHTFPAV**LQ**SSG **L**Y
1812.92	184–201	0	SLSSVVTVPSSSLG**T**QTY

**Table 4:** Full list of glycans found on all mutants.

# Structure	Type	Composition	Structure	[M-2H]^2−^	Glycosite
1a	high mannose	HexNAc(2)Hex(5)	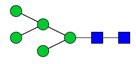	617.22	Q178N
1b	high mannose	HexNAc(2)Hex(5)	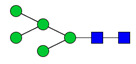	617.22	L177N Q178N L182N
2a (G0)	complex	HexNAc(4)Hex(3)	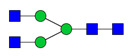	658.24	L177N Herceptin
2b (G0)	complex	HexNAc(4)Hex(3)	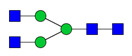	658.24	L177N
3	high mannose	HexNAc(2)Hex(6)	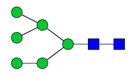	698.25	Q178N L182N
4 (G0F)	complex	HexNAc(4)Hex(3)Fuc(1)	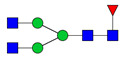	731.27	All Tmab variants
5	complex	HexNAc(5)Hex(3)	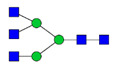	759.78	L177N
6a	hybrid	HexNAc(3)Hex(4)Man(2) Fuc(1)	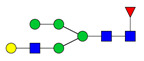	791.79	L177N
6b	hybrid	HexNAc(3)Hex(4)Man(2) Fuc(1)	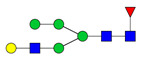	791.79	L177N
6c	hybrid	HexNAc(3)Hex(4)Man(2) Fuc(1)	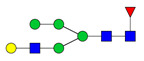	791.79	L177N
7	hybrid	HexNAc(3)Hex(5)Man(1)	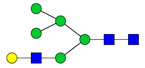	799.79	Q178N L182N
8a (G1F)	complex	HexNAc(4)Hex(3)Man(1) Fuc(1)	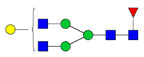	812.3	L177N
8b (G1F)	complex	HexNAc(4)Hex(3)Man(1) Fuc(1)	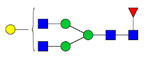	812.3	All Tmab variants
9	complex	HexNAc(5)Hex(3)Man(1)	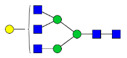	840.81	L177N
10	high mannose	HexNAc(2)Hex(8)	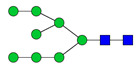	860.3	L115N
11	hybrid	HexNAc(3)Hex(4)Man(1) NeuAc(1)	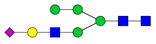	864.31	L182N
12	hybrid	HexNAc(3)Hex(5)Man(1) Fuc(1)	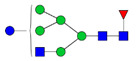	872.81	L177N
13 (G2F)	complex	HexNAc(4)Hex(3)Man(2) Fuc(1)	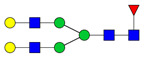	893.33	All Tmab variants
14a	complex	HexNAc(5)Hex(3)Man(1) Fuc(1)	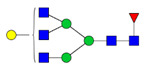	913.84	A121N L177N Q178N L182N T198N
14b	complex	HexNAc(5)Hex(3)Man(1) Fuc(1)	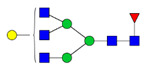	913.84	A121N L177N Q178N L182N T198N
15	high mannose	HexNAc(2)Hex(9)	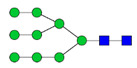	941.33	L115N
16	hybrid	HexNAc(3)Hex(5)Man(1) NeuAc(1)	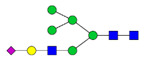	944.84	A121N L177N L182N T198N
17a	complex	HexNAc(4)Hex(3)Man(2) NeuAc(1)	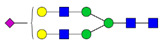	965.84	L177N L182N
17b	complex	HexNAc(4)Hex(3)Man(2) NeuAc(1)	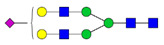	965.84	L177N
18	complex	HexNAc(5)Hex(3)Man(1) Fuc(2)	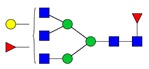	986.87	A121N L177N
19a	complex	HexNAc(5)Hex(3)Man(2) Fuc(1)	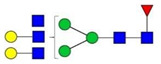	994.87	L115N A121N L177N Q178N L182N T198N
19b	complex	HexNAc(5)Hex(3)Man(2) Fuc(1)	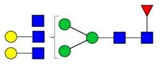	994.87	L177N
20a	complex	HexNAc(4)Hex(3)Man(2) Fuc(1)NeuAc(1)	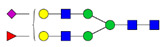	1038.87	L115N A121N L177N Q178N L182N T198N
20b	complex	HexNAc(4)Hex(3)Man(2) NeuAc(1)	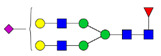	1038.87	A121N L177N Q178N L182N T198N
21a	complex	HexNAc(5)Hex(3)Man(1) NeuAc(1)	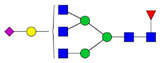	1059.39	A121N L177N T198N
21b	complex	HexNAc(5)Hex(3)Man(1) NeuAc(1)	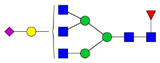	1059.39	L177N
21c	complex	HexNAc(5)Hex(3)Man(1) NeuAc(1)	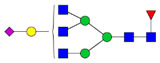	1059.39	L177N

**Table 5:** Binding constants of the Tmab variants to HER2 and FcγR1A. Listed are the on rate (***Ka***), off rate (***Kd***) and the calculated affinity constant (***K_D_***) for each data set.

Protein	HER2			FcγR1A		
	*K_a_* (M^−1^ × s^−1^)	*K_d_* (s^−1^)	*K_D_* (pM)	*K_a_* (M^−1^ × s^−1^)	*K_d_* (s^−1^)	*K_D_* (nM)
Herceptin	1.3 × 10^6^	2.4 × 10^−5^	18	2.4 × 10^5^	1.1 × 10^−3^	4.7
HEK Trastuzumab	7.9 × 10^5^	2.7 × 10^−5^	34	1.1 × 10^5^	1.0 × 10^−3^	9.9
L115N	2.4 × 10^5^	2.7 × 10^−4^	1130	7.8 × 10^5^	5.0 × 10^−4^	6.4
L115N	2.4 × 10^5^	2.8 × 10^−4^	1160	1.1 × 10^5^	4.0 × 10^−4^	3.7
A121N	3.8 × 10^5^	3.0 × 10^−4^	790	1.7 × 10^5^	8.3 × 10^−4^	4.8
A121N	4.1 × 10^5^	2.7 × 10^−4^	640	2.0 × 10^5^	9.8 × 10^−4^	4.8
Q160N	2.2 × 10^5^	4.3 × 10^−5^	198	1.2 × 10^5^	7.6 × 10^−4^	6.5
Q160N	Responses to low for accurate fit			1.1 × 10^5^	7.6 × 10^−4^	6.7
L177N	4.0 × 10^5^	1.9 × 10^−4^	480	2.5 × 10^5^	1.4 × 10^−3^	5.6
L177N	4.5 × 10^5^	2.4 × 10^−4^	530	2.6 × 10^5^	1.5 × 10^−3^	5.6
Q178N	3.9 × 10^5^	1.8 × 10^−4^	480	3.7 × 10^5^	9.6 × 10^−4^	2.6
Q178N	3.8 × 10^5^	8.7 × 10^−5^	230	3.8 × 10^5^	9.6 × 10^−4^	2.5
L182N	3.6 × 10^5^	3.9 × 10^−5^	109	1.3 × 10^5^	1.1 × 10^-3^	8.2
L182N	5.6 × 10^5^	6.4 × 10^−5^	110	1.8 × 10^5^	1.5 × 10^−3^	8.1
T198N	2.5 × 10^5^	2.3 × 10^−4^	920	2.4 × 10^5^	8.8 × 10^−4^	3.8
T198N	4.6 × 10^5^	2.5 × 10^−4^	550	2.4 × 10^5^	9.0 × 10^−4^	3.8

**Table 6:** Binding constants of the Tmab variants to FcγR2B and FcγR3A. Listed are the *K_D_* and the SD of the fit for each experiment where data were able to be obtained.

Protein	FcγR2B	FcγR3A
	*K_D_* (nM)	*K_D_* (nM)
Herceptin	666 ± 63	267 ± 34
HEK Trastuzumab	686 ± 127	433 ± 65
L115N	338 ±100	394 ± 37
L115N	269 ± 160	301 ± 40
A121N	3142 ± 340	295 ± 17
A121N	3743 ± 720	268 ± 14
Q160N	143 ± 43	596 ± 100
Q160N	poor data set	535 ± 67
L177N	1275 ± 120	535 ± 79
L177N	992 ± 230	551 ± 71
Q178N	Responses too low for accurate fit	239 ± 25
Q178N	485 ± 240	217 ± 30
L182N	No observable binding	ND
L182N		
T198N	Responses too low for accurate fit	ND
T198N	666 ± 63	

ND: not determined.

**Table 7:** *T_m_* and *T_agg_* of Herceptin, HEK Trastuzumab and variants with the exception of Q160N.

Protein	*T_m_*1 (°C)	*T_m_*2 (°C)	*T_agg_* @ 266 nm
Herceptin	68.4 ± 0.2	82.5 ± 0.1	81.2 ± 0.1
Tmab	68.2 ± 0.1	83.1 ± 0.3	85.2 ± 0.1
L115N	73.1 ± 0.7	84.3 ± 0.4	91.2 ± 0.3
A121N	68.8 ± 0.2	80.7 ± 0.5	85.2 ± 0.1
L177N	71.2 ± 0.3	79.1 ± 0.3	85.1 ± 0.1
Q160N	ND	ND	ND
Q178N	69.1 ± 0.2	81.1 ± 0.4	85.5 ± 0.1
L182N	73.1 ± 1.9	81.0 ± 0.3	86.1 ± 0.1
T198N	67.9 ± 0.4	83.3 ± 0.4	87.8 ± 0.1

ND: not determined.

## Data Availability

Research data and related materials are retained according to the Research Data Management Policy and Research Code of Conduct of the University of Sydney and are stored on University-managed storage infrastructure. Research data can be made available for re-use by other researchers, unless this is prevented by the requirements of legislation or University policy, or ethical, contractual or confidentiality obligations; where applicable.

## Supplementary Materials

The following are available online at https://www.mdpi.com/article/10.3390/pharmaceutics13111747/s1, Figure S1. Reducing SDS-PAGE of the trastuzumab variants expressed in HEK-293 cells under the exact same expression conditions for assessment of glycan attachment. Commercial Herceptin (Herc) and the HEK Trastuzumab (HEK Tmab, non-mutated trastuzumab produced in-house) were run together with the mutants to compare electrophoretic mobility shifts in the ~50 kDa band corresponding to the heavy chain. Figure S2. Full MS spectra of released glycans from HEK Trastuzumab. Figure S3. Full MS spectra of released glycans from Herceptin. Figure S4. Full MS spectra of released glycans from L115N. Figure S5. Full MS spectra of released glycans from A121N. Figure S6. Full MS spectra of released glycans from Q160N. Figure S7. Full MS spectra of released glycans from L177N. Figure S8. Full MS spectra of released glycans from Q178N. Figure S9. Full MS spectra of released glycans from L182N. Figure S10. Full MS spectra of released glycans from T198N. Figure S11. MS2 spectra of glycan 1 (Table 4). Figure S12. MS2 spectra of glycan 2 (Table 4). Figure S13. MS2 spectra of glycan 3 (Table 4). Figure S14. MS2 spectra of glycan 4 (Table 4). Figure S15. MS2 spectra of glycan 5 (Table 4). Figure S16. MS2 spectra of glycan 6 (Table 4). Figure S17. MS2 spectra of glycan 7 (Table 4). Figure S18. MS2 spectra of glycan 8 (Table 4). Figure S19. MS2 spectra of glycan 9 (Table 4). Figure S20. MS2 spectra of glycan 10 (Table 4). Figure S21. MS2 spectra of glycan 11 (Table 4). Figure S22. MS2 spectra of glycan 12 (Table 4). Figure S23. MS2 spectra of glycan 13 (Table 4). Figure S24. MS2 spectra of glycan 14 (Table 4). Figure S25. MS2 spectra of glycan 15 (Table 4). Figure S26. MS2 spectra of glycan 16 (Table 4). Figure S27. MS2 spectra of glycan 17 (Table 4). Figure S28. MS2 spectra of glycan 18 (Table 4). Figure S29. MS2 spectra of glycan 19 (Table 4). Figure S30. MS2 spectra of glycan 20 (Table 4). Figure S31. MS2 spectra of glycan 21 (Table 4). Figure S32. CID spectrum of the chymotryptic peptide DYFPEPVTVSWNSGALTSGVHTFPAVLQSSGLY from the L115N mutant containing amino acid positions 177, 178 and 182. Figure S33. CID spectrum of the chymotryptic peptide DYFPEPVTVSWNSGALTSGVHTFPAVLQSSGLY from the A121N mutant amino acid positions 177, 178 and 182. Figure S34. CID spectrum of the chymotryptic peptide SGALTSGVHTFPAVLQSSGLY from Tmab WT amino acid positions 177, 178 and 182. Figure S35. CID spectrum of the chymotryptic peptide VDNALQSGNSNESVTEQDSKDSTYSLSSTLTLSK from the Q160N mutant confirming the mutation at position 160. Figure S36. Analysed SPR sensorgrams (black trace) of single cycle kinetic assays of Tmab variants to surface captured HER2. Blue trace is the data fit. Figure S37. Analysed SPR sensorgrams (black trace) of single cycle kinetic assays of Tmab variants to surface captured FcγR1A. Blue trace is the data fit. Figure S38. Left panel: SPR sensorgrams of single cycle kinetic assays of Tmab variants to surface captured FcγR2A. Right Panel: Fits to equilibrium responses from the sensorgrams. Figure S39. Left panel: SPR sensorgrams of single cycle kinetic assays of Tmab variants to surface captured FcγR3A. Right Panel: Fits to equilibrium responses from the sensorgrams.
