# Supplementary material for: Glycan Profile Analysis of Engineered Trastuzumab with Rationally Added Glycosylation Sequons Presents Significantly Increased Glycan Complexity
Source: Pharmaceutics. 2021 Oct 20;13(11):1747. doi: 10.3390/pharmaceutics13111747 (PMC8620955; doi:10.3390/pharmaceutics13111747)
Supplement: Supplementary file 1 [file pharmaceutics-13-01747-s001.zip › pharmaceutics-1399290-supplementary.pdf]

pharmaceutics

MDPI

Supplementary Materials: Glycan Profile Analysis of Engineered Trastuzumab with Rationally Added Glycosylation Sequons Presents Significantly Increased Glycan Complexity

Esteban Cruz, Vicki Sifniotis, Zeynep Sumer-Bayraktar, Mouhamad Reslan, Lorna Wilkinson-White, Stuart Cordwell and Veysel Kayser

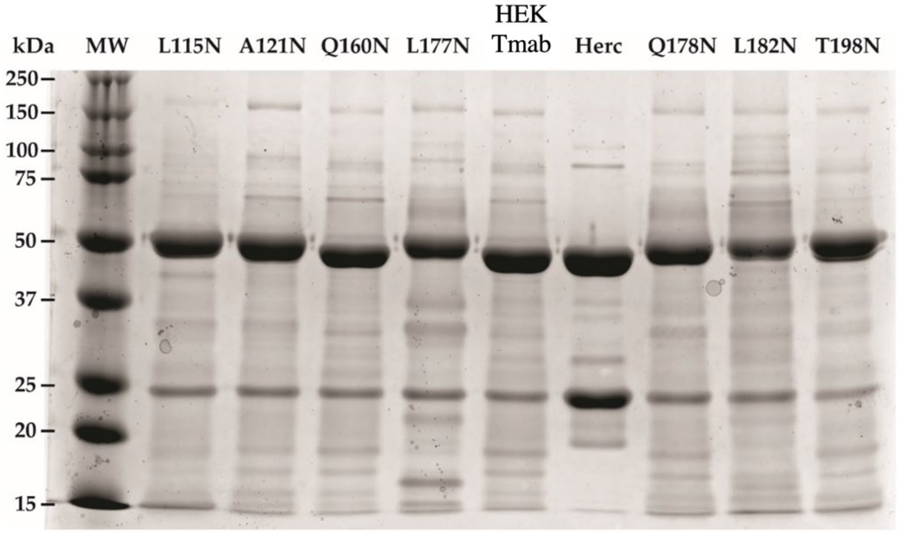

Figure S1. Reducing SDS-PAGE of the trastuzumab variants expressed in HEK-293 cells under the exact same expression conditions for assessment of glycan attachment. Commercial Herceptin (Herc) and the HEK Trastuzumab (HEK Tmab, non-mutated trastuzumab produced in-house) were run together with the mutants to compare electrophoretic mobility shifts in the ~50 kDa band corresponding to the heavy chain.

Full MS spectra of the Tmab variants

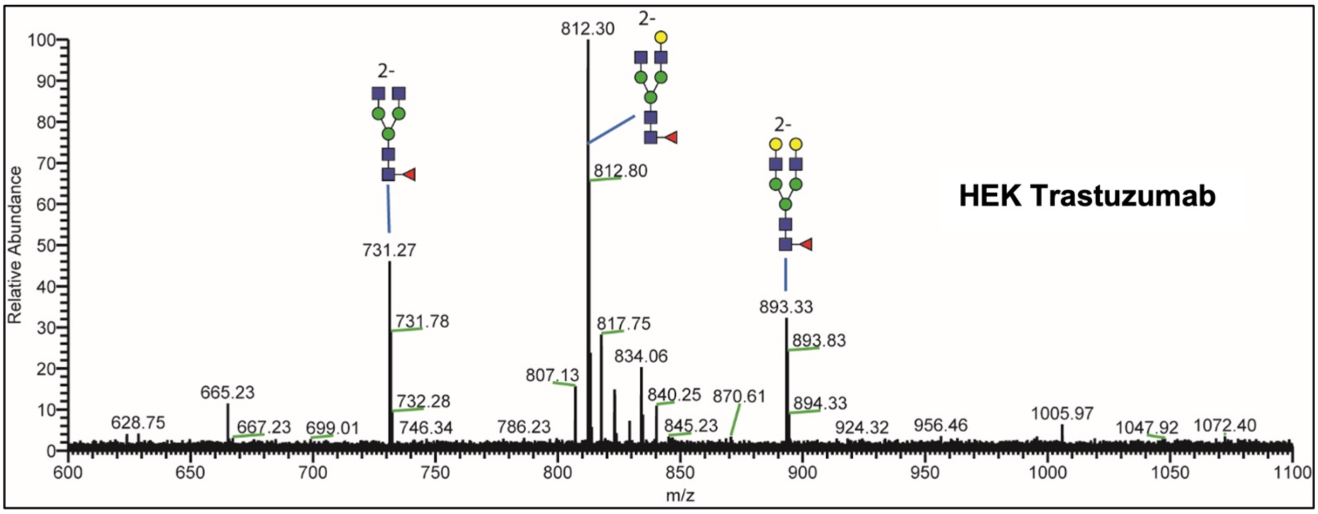

Figure S2. Full MS spectra of released glycans from HEK Trastuzumab.

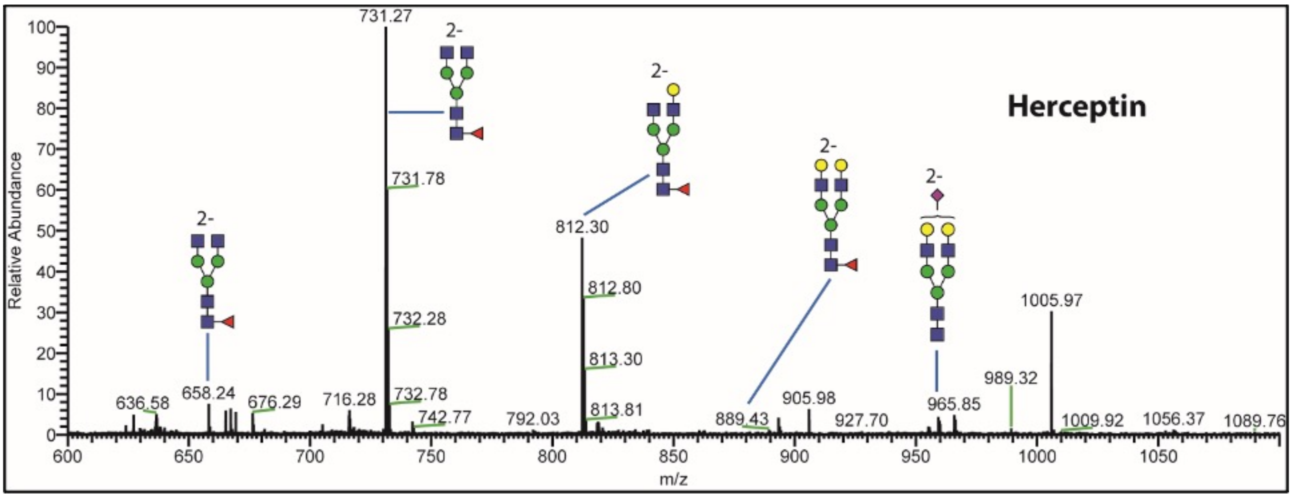

Figure S3. Full MS spectra of released glycans from Herceptin.

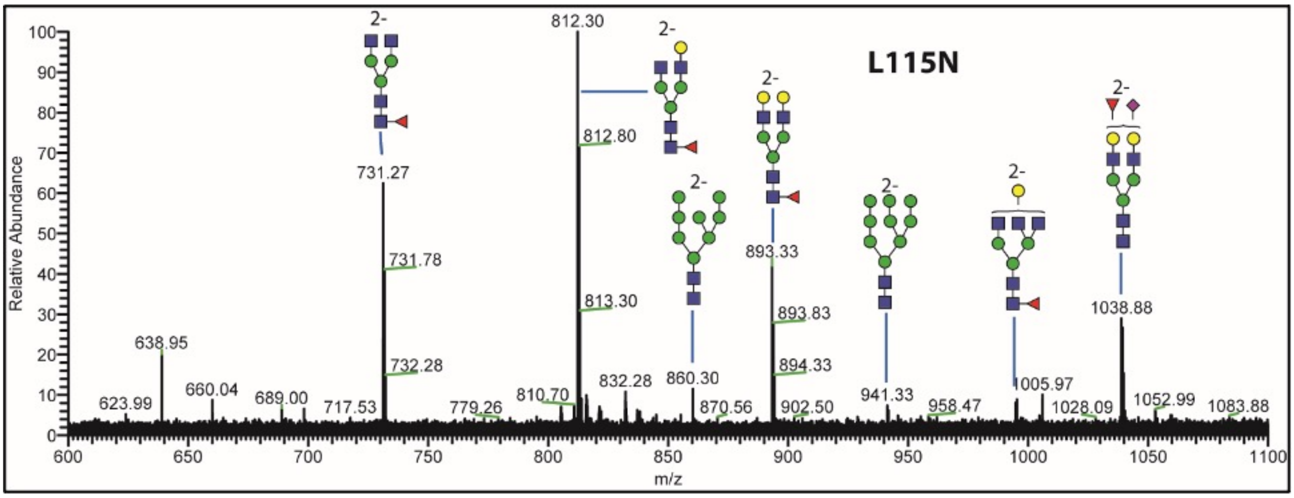

Figure S4. Full MS spectra of released glycans from L115N.

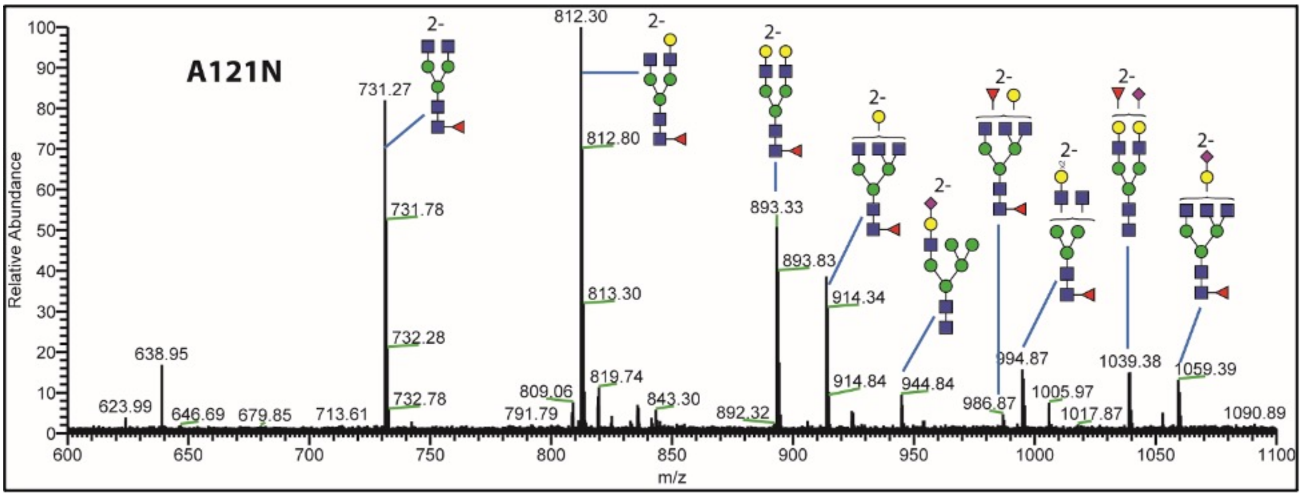

Figure S5. Full MS spectra of released glycans from A121N.

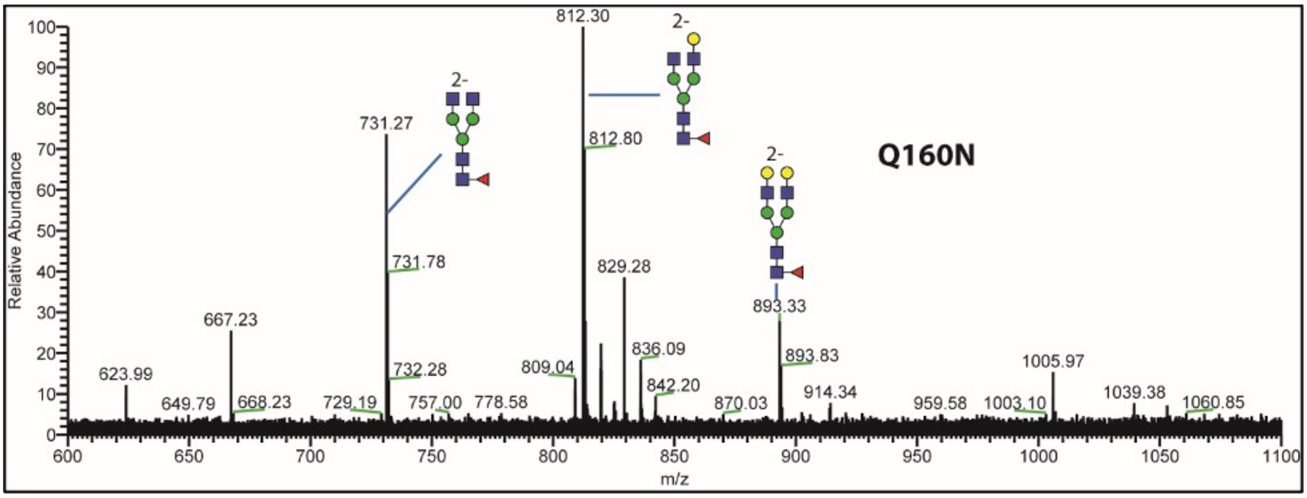

Figure S6. Full MS spectra of released glycans from Q160N.

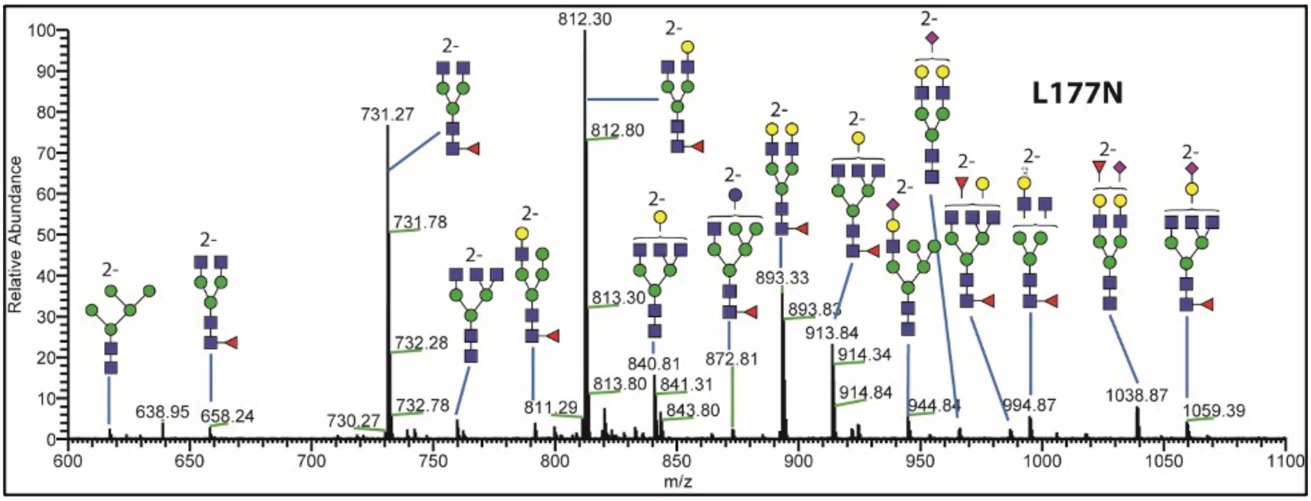

Figure S7. Full MS spectra of released glycans from L177N.

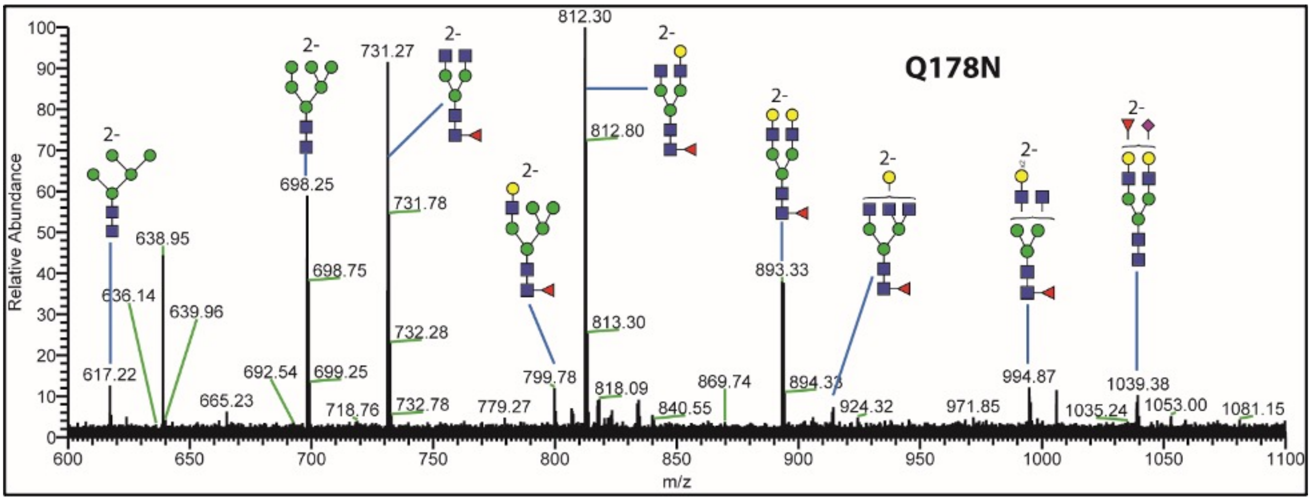

Figure S8. Full MS spectra of released glycans from Q178N.

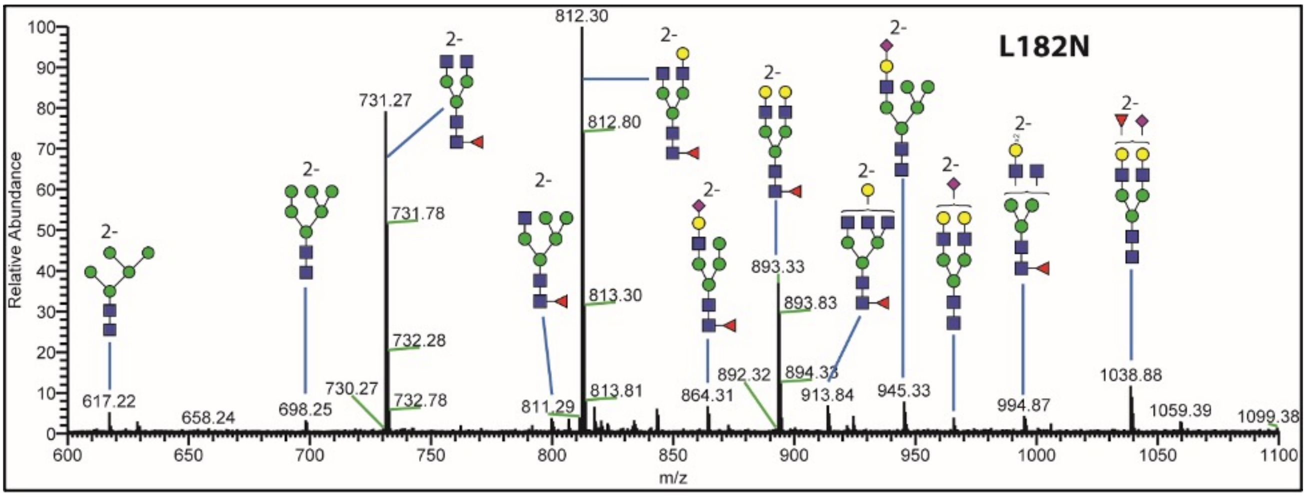

Figure S9. Full MS spectra of released glycans from L182N.

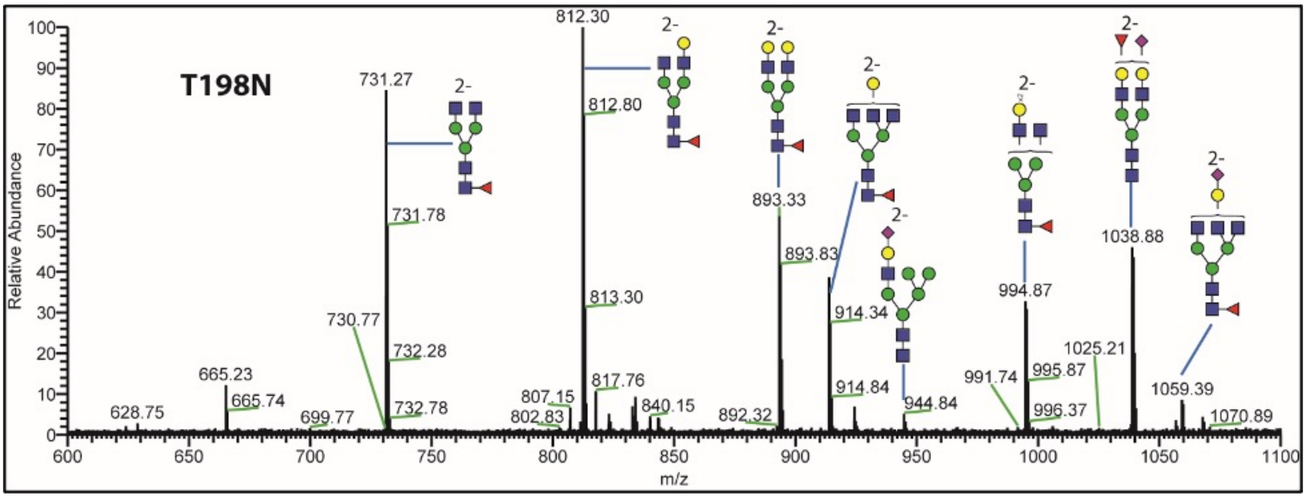

Figure S10. Full MS spectra of released glycans from T198N.

MS2 spectra

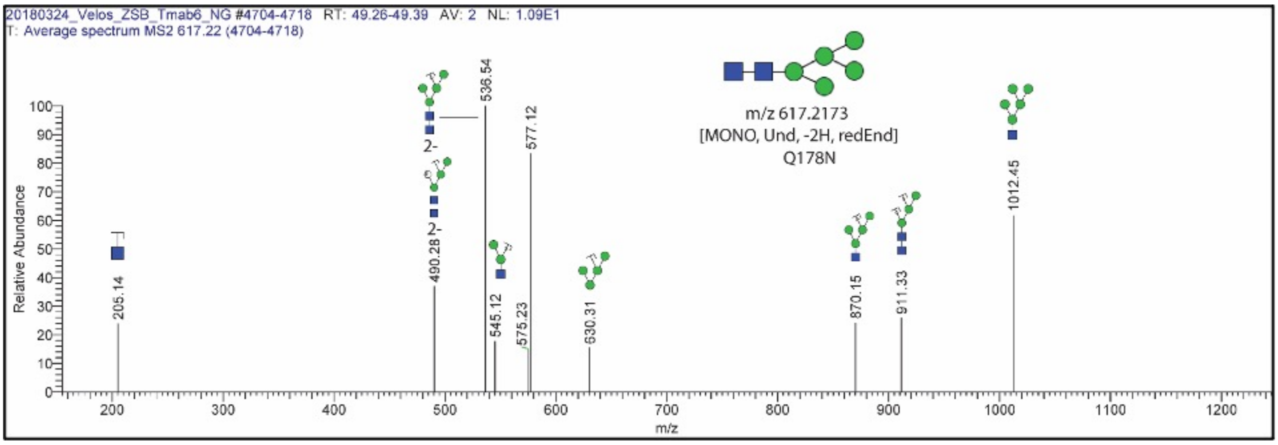

Figure S11. MS2 spectra of glycan 1 (Table 4).

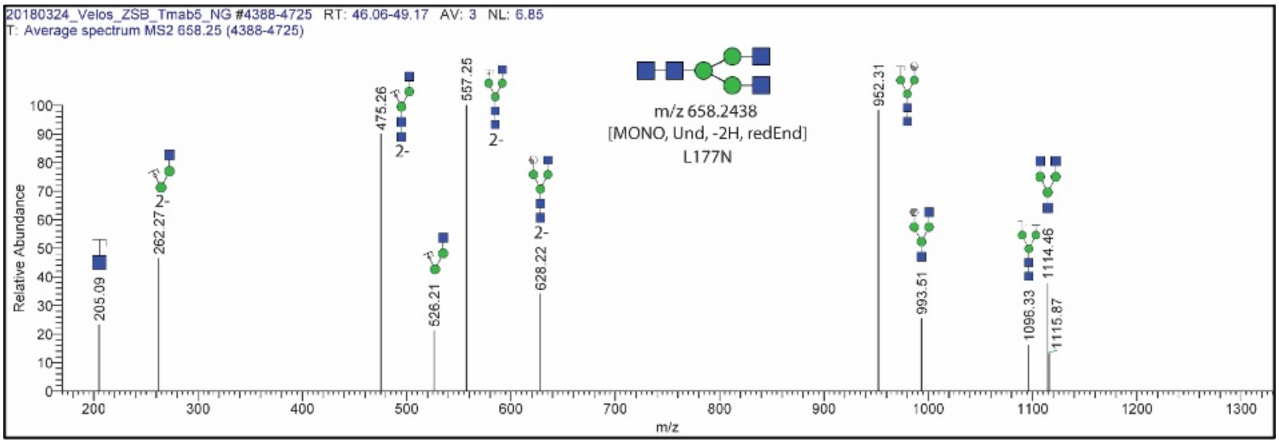

Figure S12. MS2 spectra of glycan 2 (Table 4).

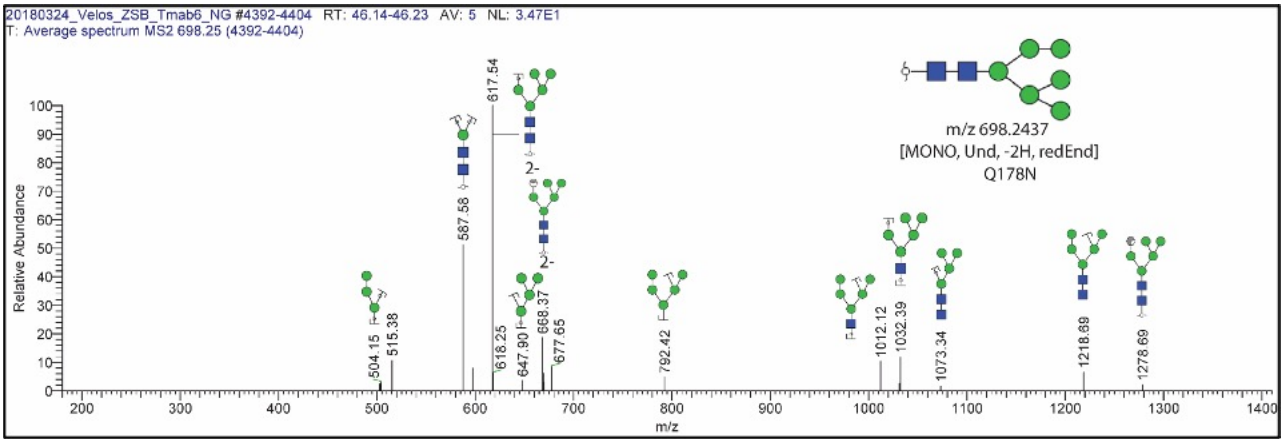

Figure S13. MS2 spectra of glycan 3 (Table 4).

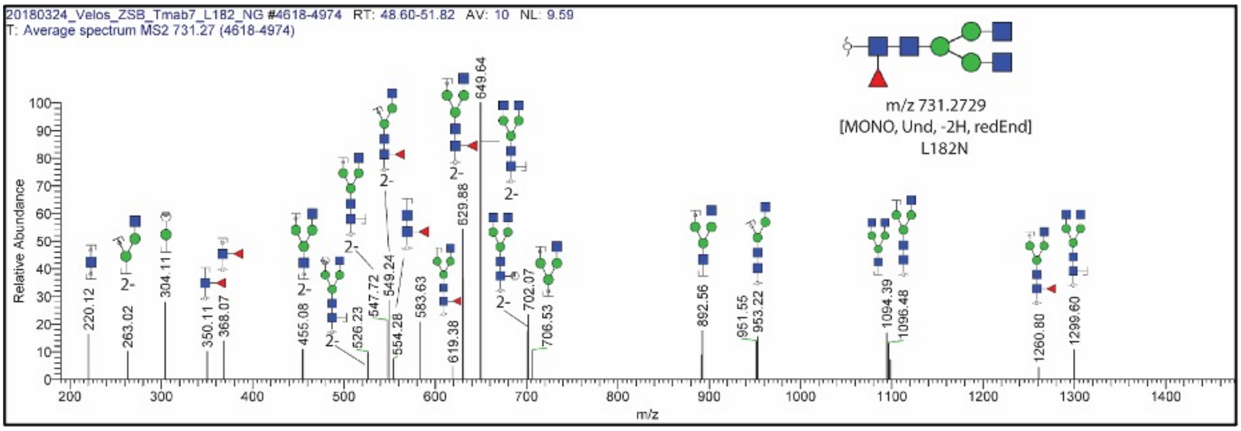

Figure S14. MS2 spectra of glycan 4 (Table 4).

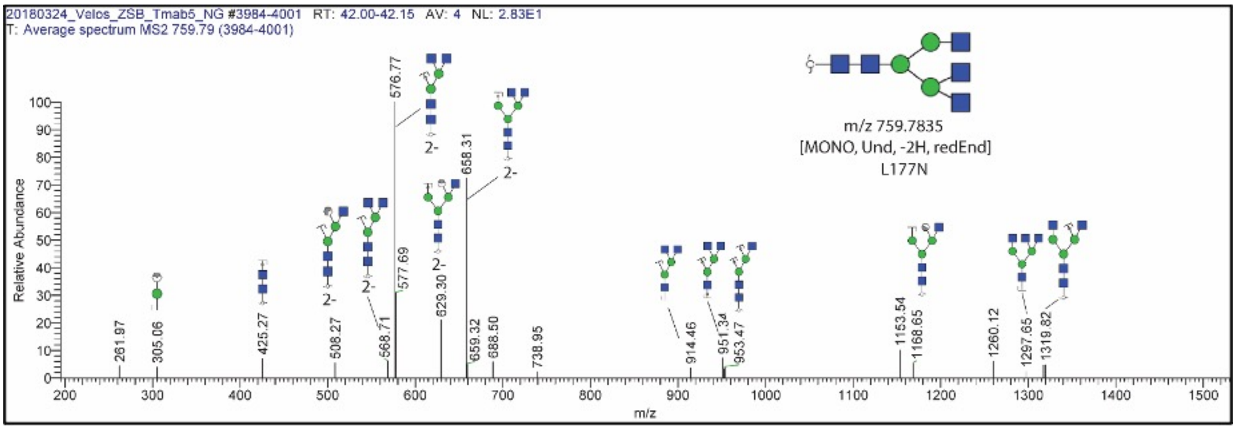

Figure S15. MS2 spectra of glycan 5 (Table 4).

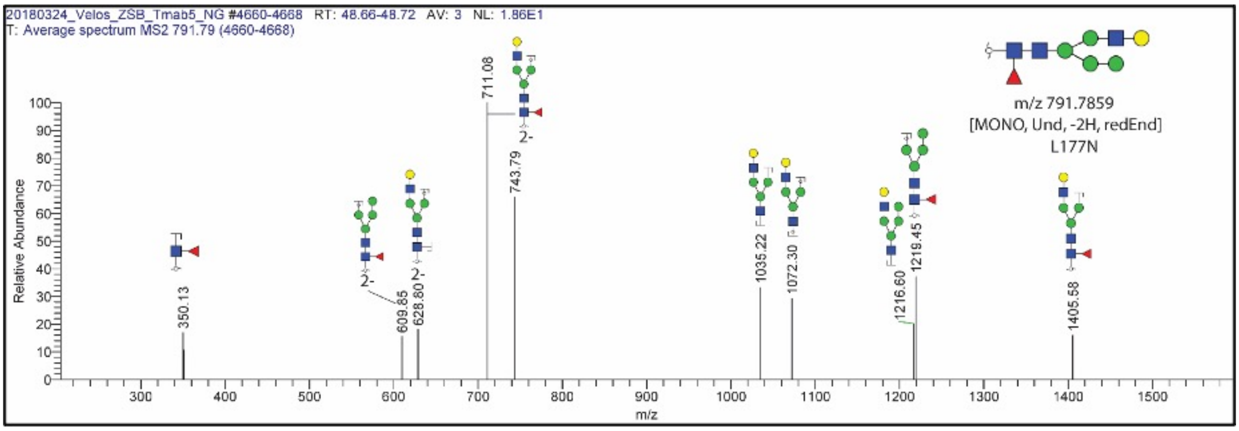

Figure S16. MS2 spectra of glycan 6 (Table 4).

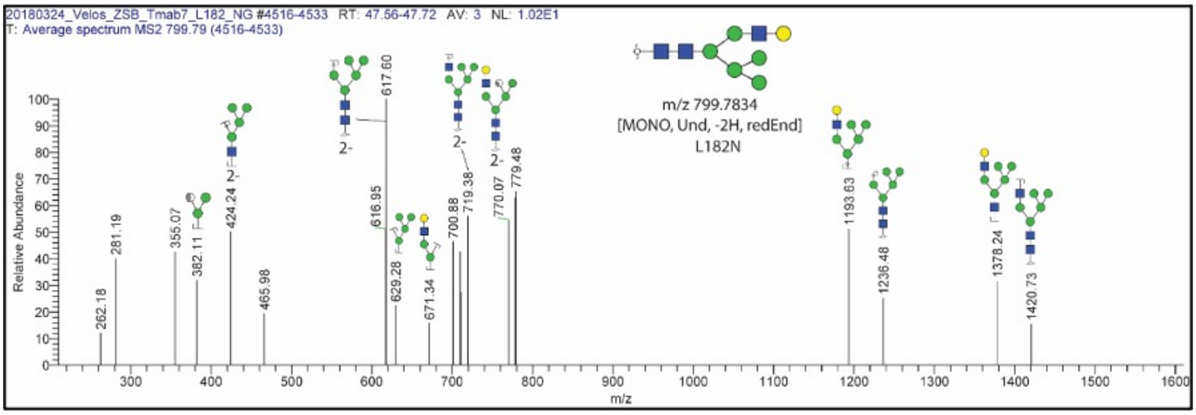

Figure S17. MS2 spectra of glycan 7 (Table 4).

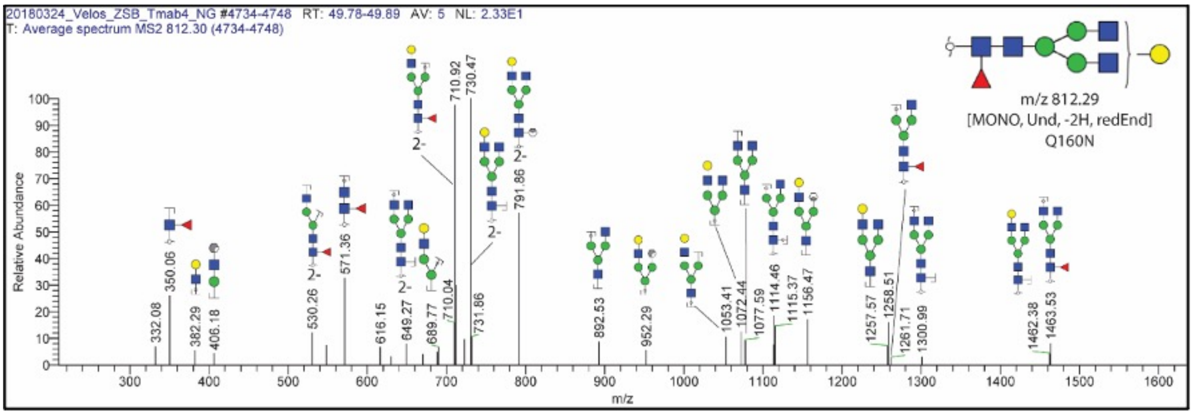

Figure S18. MS2 spectra of glycan 8 (Table 4).

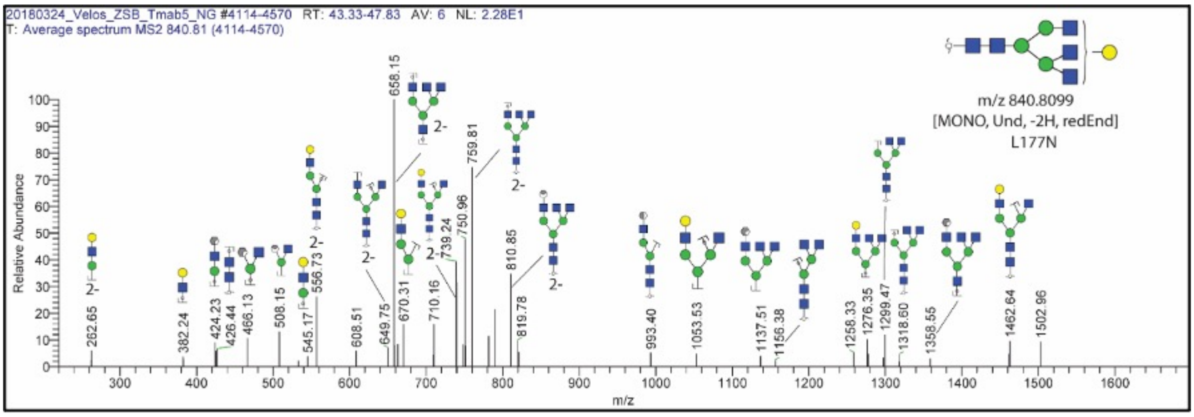

Figure S19. MS2 spectra of glycan 9 (Table 4).

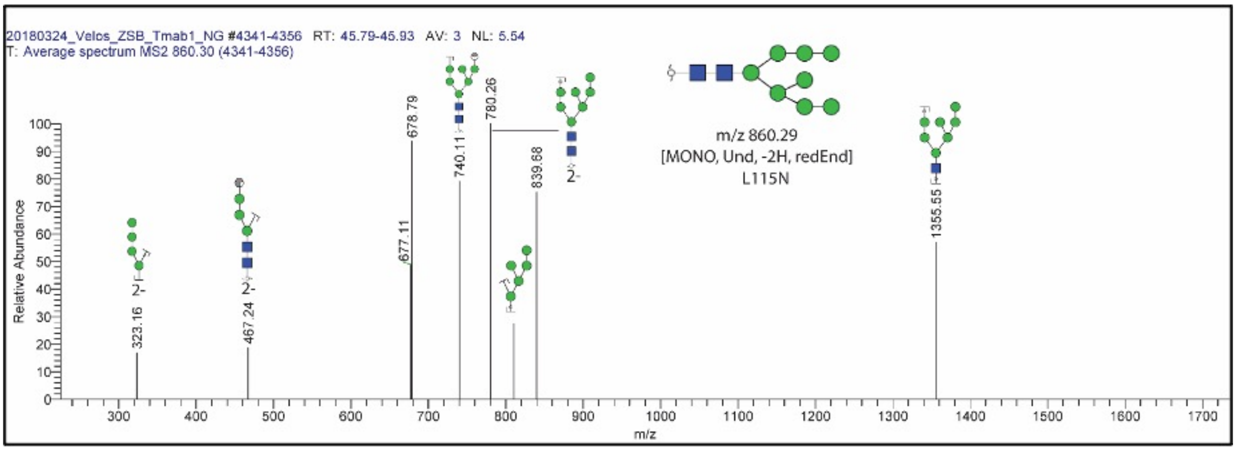

Figure S20. MS2 spectra of glycan 10 (Table 4).

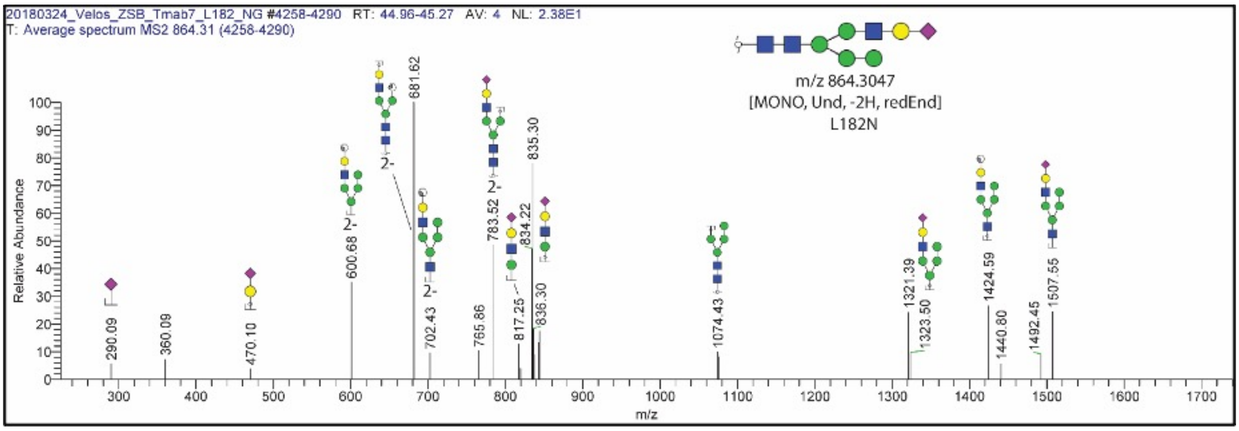

Figure S21. MS2 spectra of glycan 11 (Table 4).

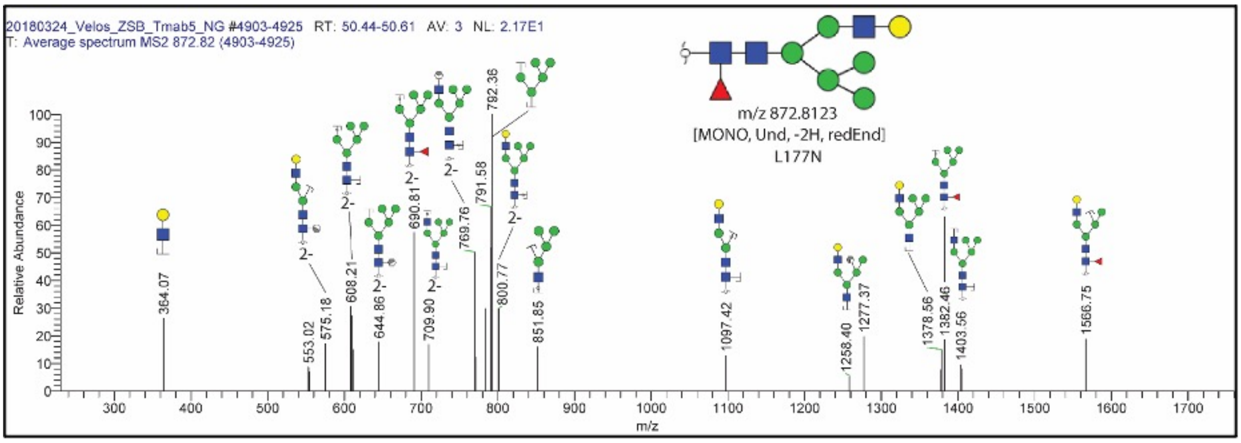

Figure S22. MS2 spectra of glycan 12 (Table 4).

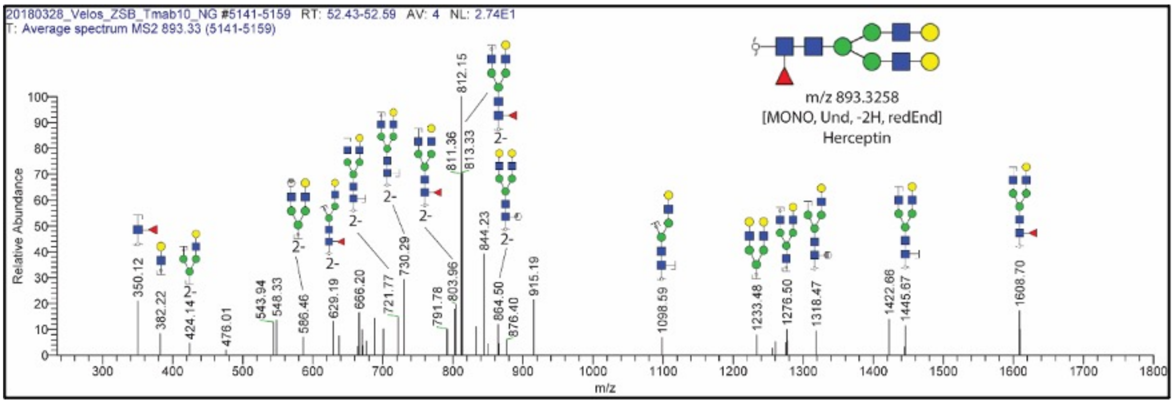

Figure S23. MS2 spectra of glycan 13 (Table 4).

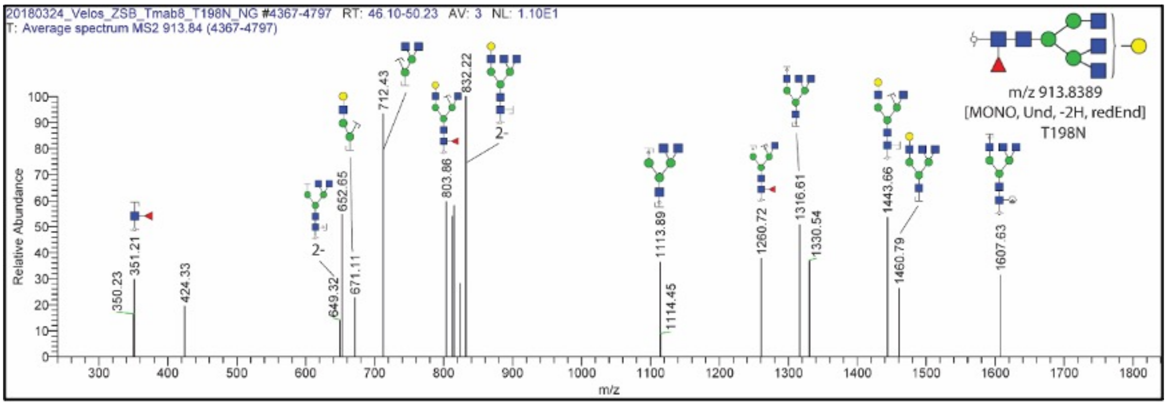

Figure S24. MS2 spectra of glycan 14 (Table 4).

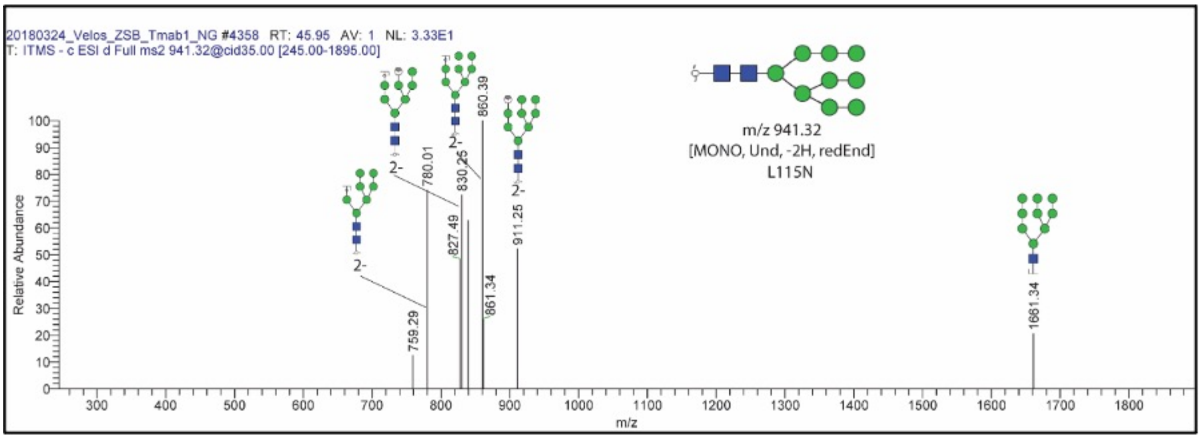

Figure S25. MS2 spectra of glycan 15 (Table 4).

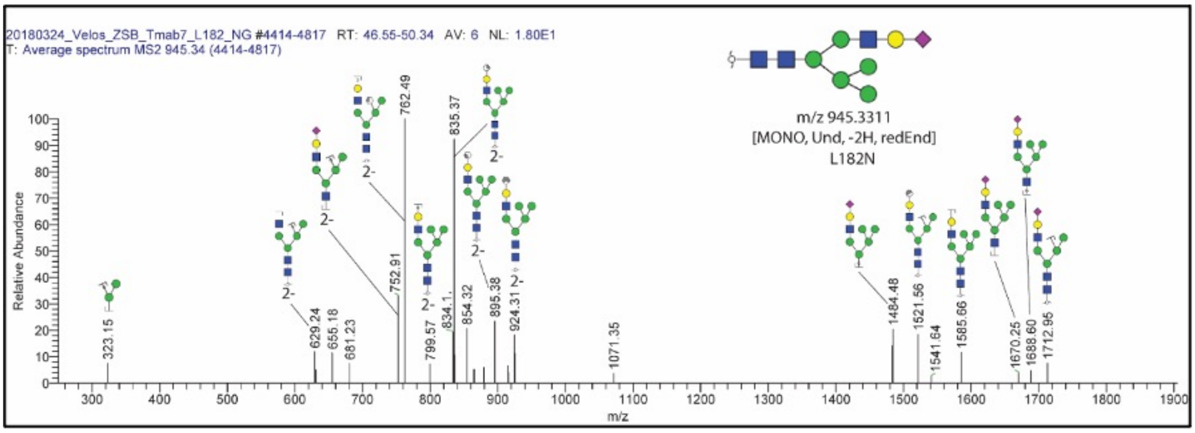

Figure S26. MS2 spectra of glycan 16 (Table 4).

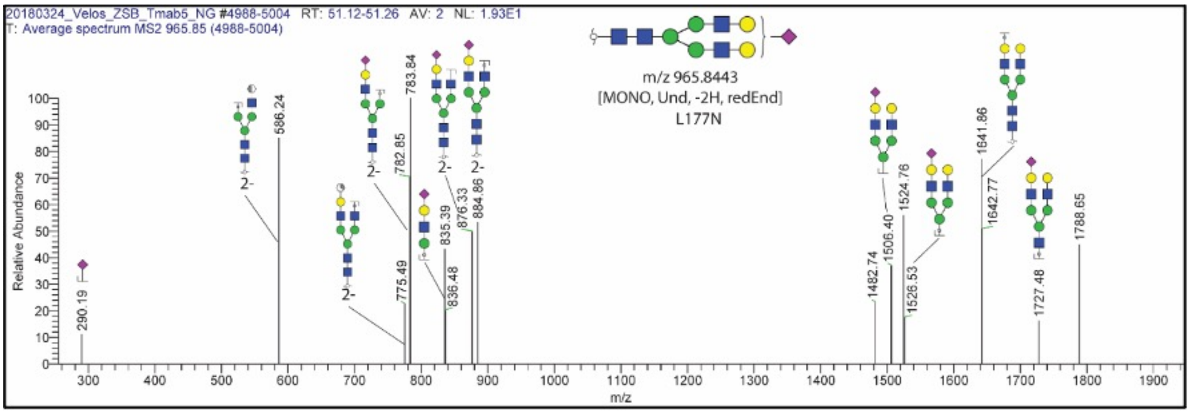

Figure S27. MS2 spectra of glycan 17 (Table 4).

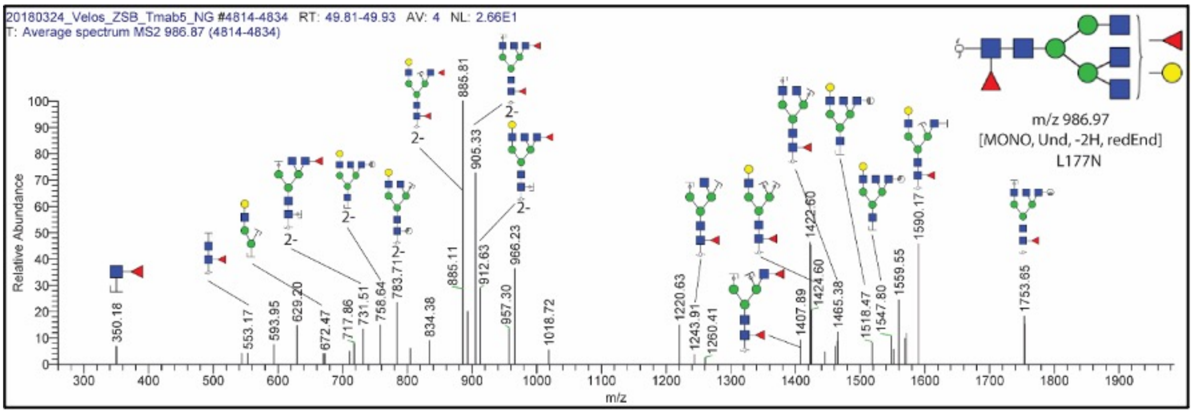

Figure S28. MS2 spectra of glycan 18 (Table 4).

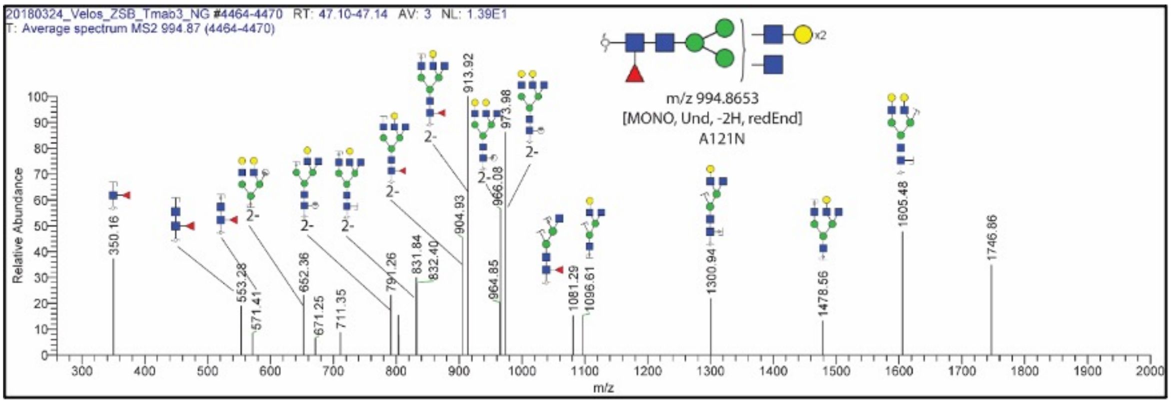

Figure S29. MS2 spectra of glycan 19 (Table 4).

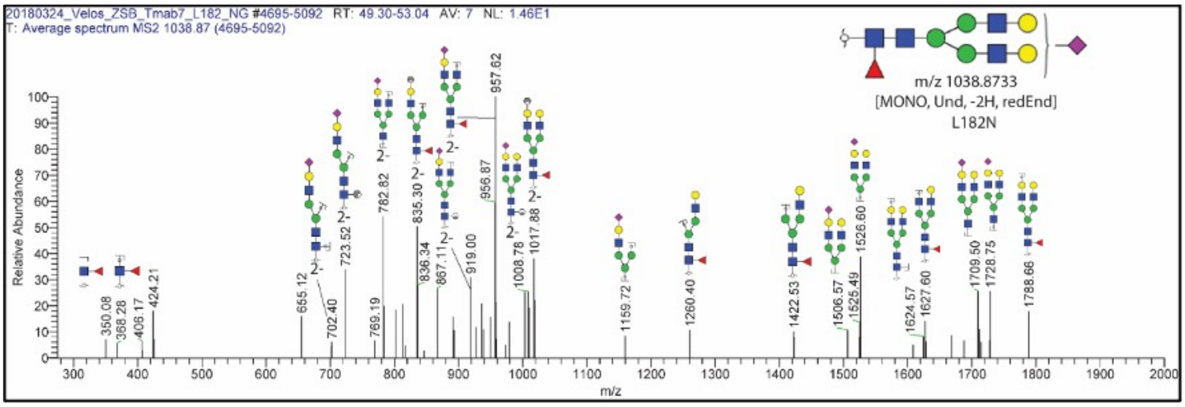

Figure S30. MS2 spectra of glycan 20 (Table 4).

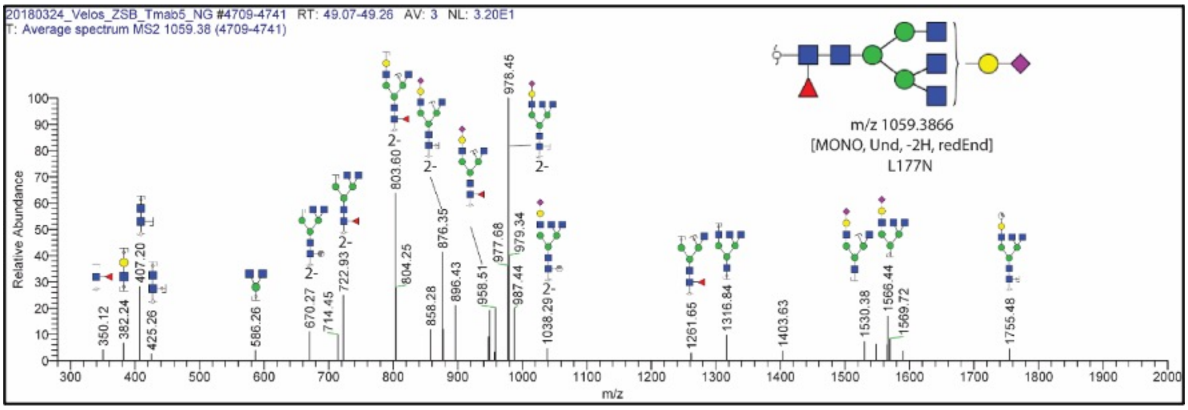

Figure S31. MS2 spectra of glycan 21 (Table 4).

Glycopeptide spectra

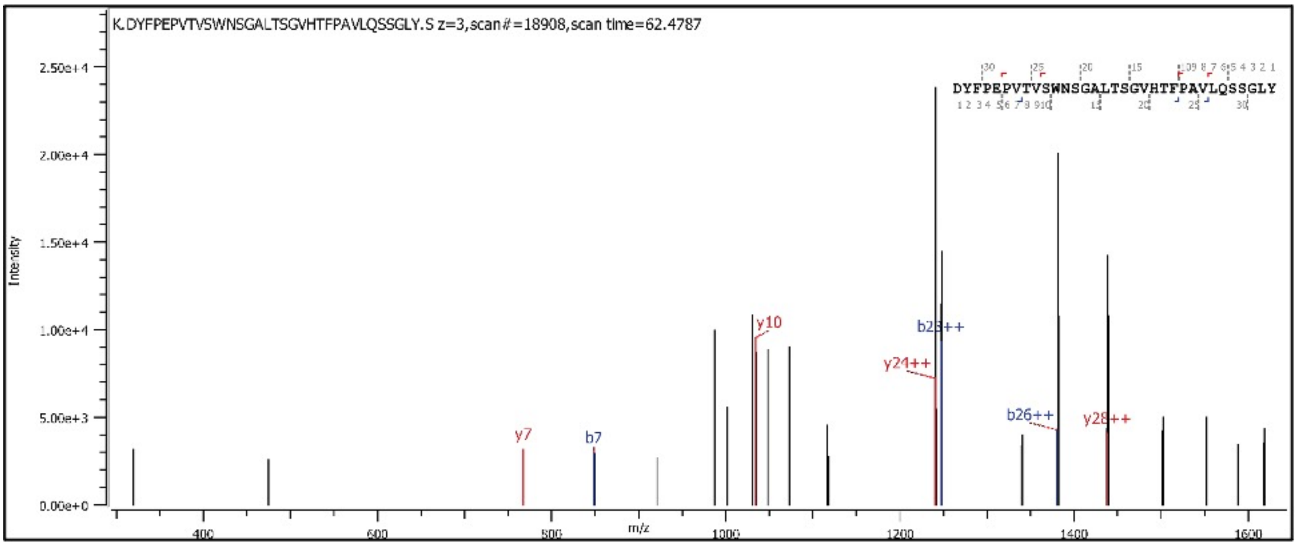

Figure S32. CID spectrum of the chymotryptic peptide DYFPEPVTVSWNSGALTSGVHTFPAVLQSSGLY from the L115N mutant containing amino acid positions 177, 178 and 182.

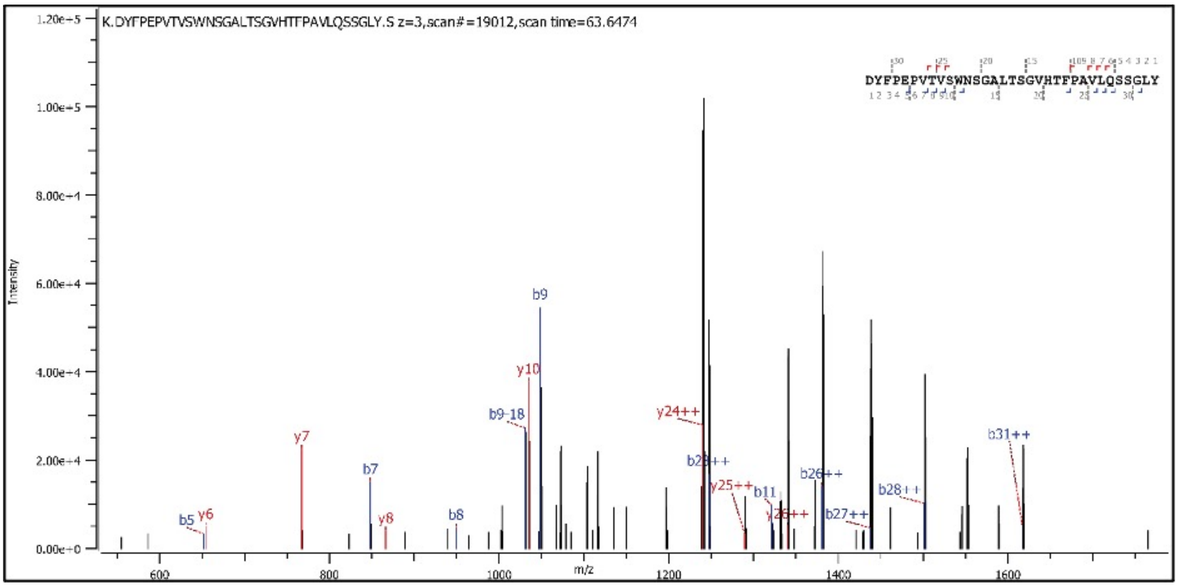

Figure S33. CID spectrum of the chymotryptic peptide DYFPEPVTVSWNSGALTSGVHTFPAVLQSSGLY from the A121N mutant amino acid positions 177, 178 and 182.

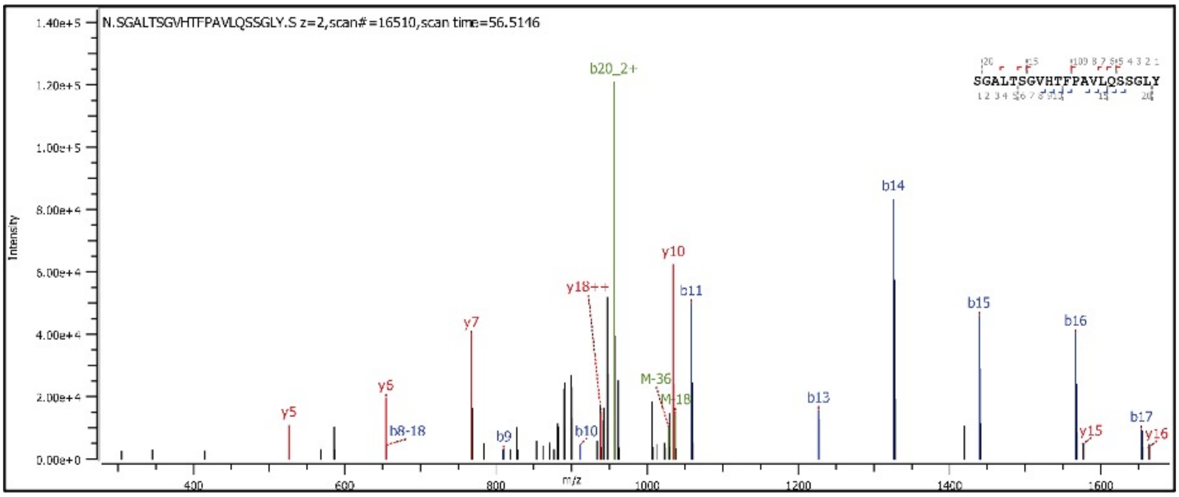

Figure S34. CID spectrum of the chymotryptic peptide SGALTSGVHTFPAVLQSSGLY from Tmab WT amino acid positions 177, 178 and 182.

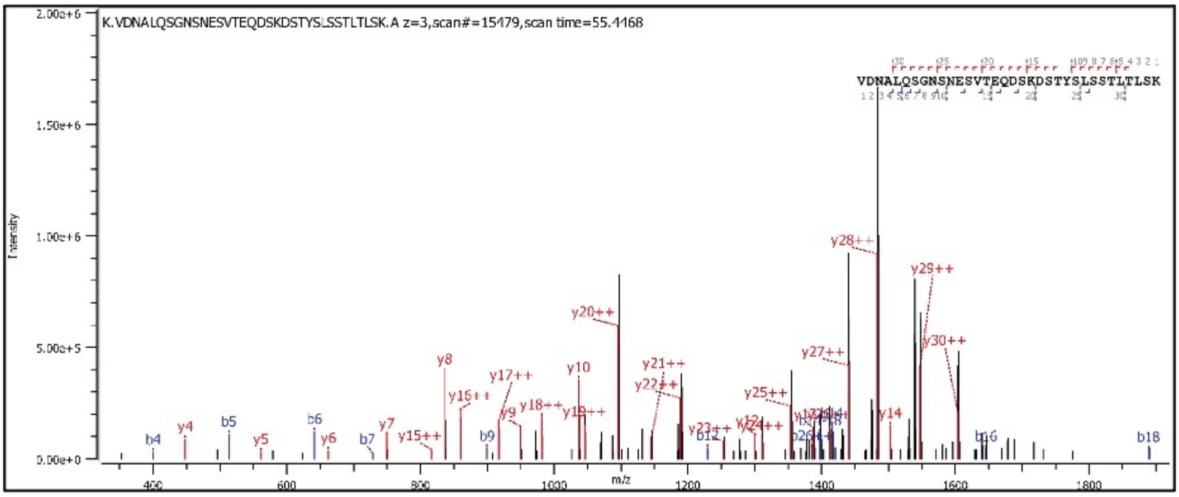

Figure S35. CID spectrum of the chymotryptic peptide VDNALQSGNSNESVTEQDSKDSTYSLSSTLTLSK from the Q160N mutant confirming the mutation at position 160.

SPR sensorgrams

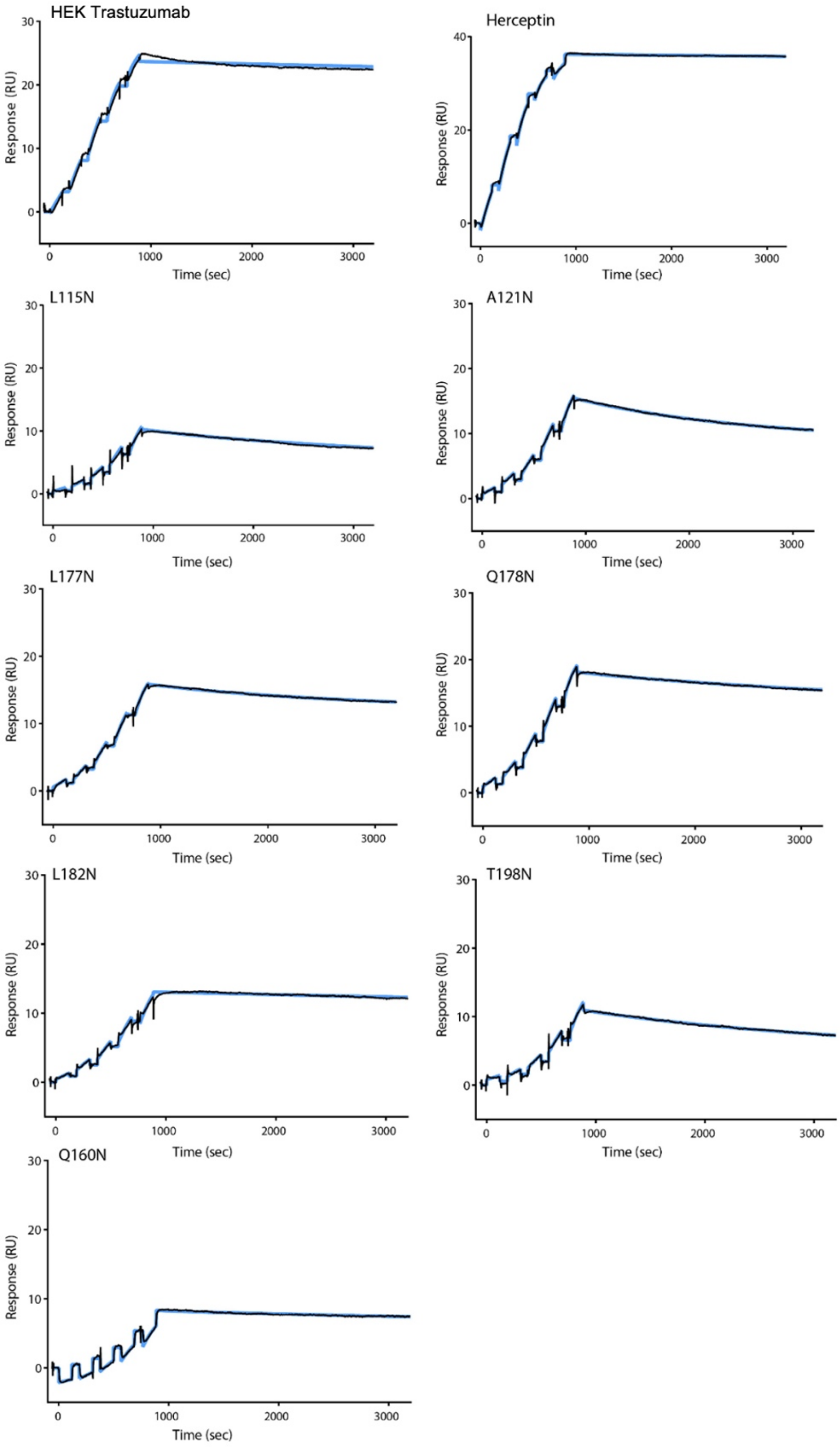

Figure S36. Analysed SPR sensorgrams (black trace) of single cycle kinetic assays of Tmab variants to surface captured HER2. Blue trace is the data fit.

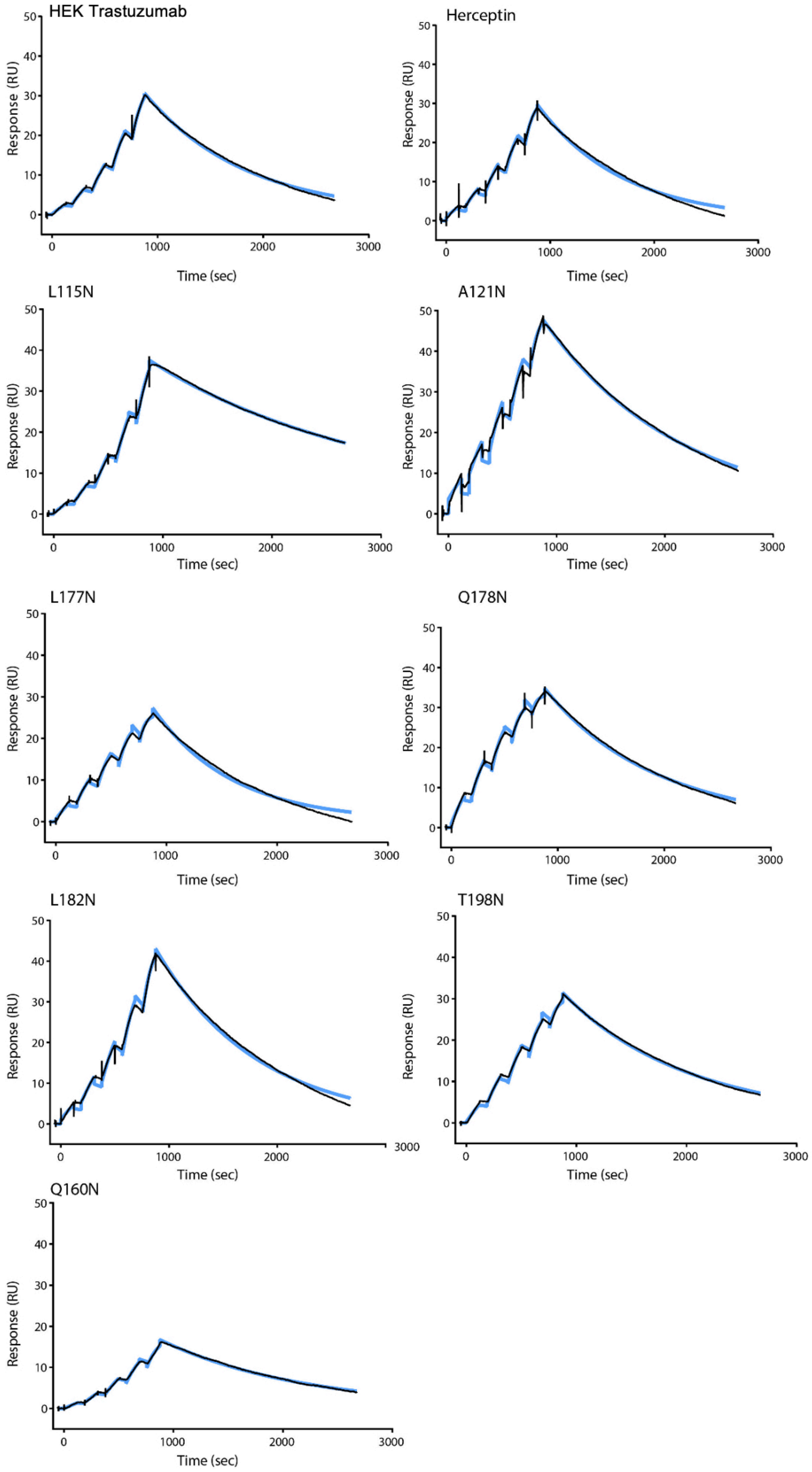

Figure S37. Analysed SPR sensorgrams (black trace) of single cycle kinetic assays of Tmab variants to surface captured FcγR1A. Blue trace is the data fit.

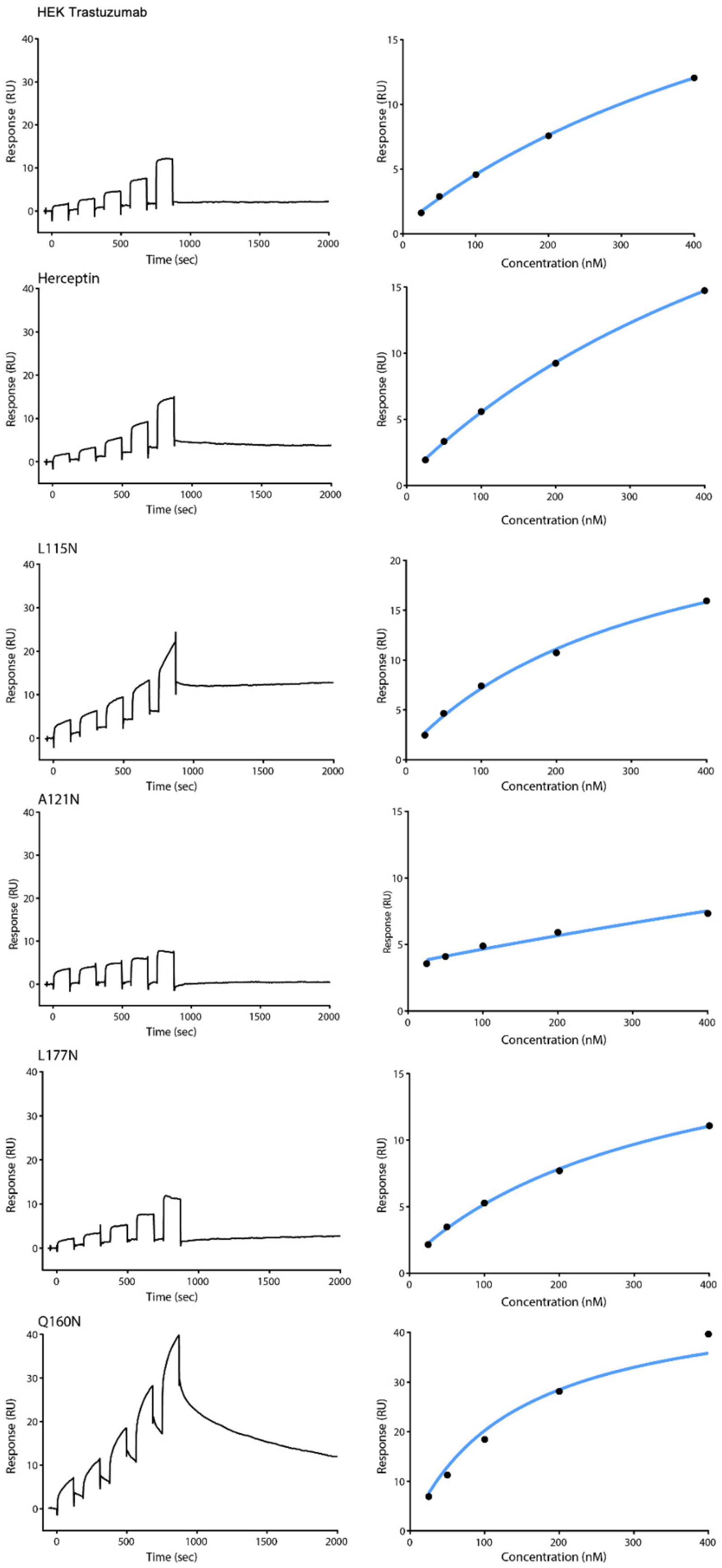

Figure S38. Left panel: SPR sensorgrams of single cycle kinetic assays of Tmab variants to surface captured FcγR2A. Right Panel: Fits to equilibrium responses from the sensorgrams.

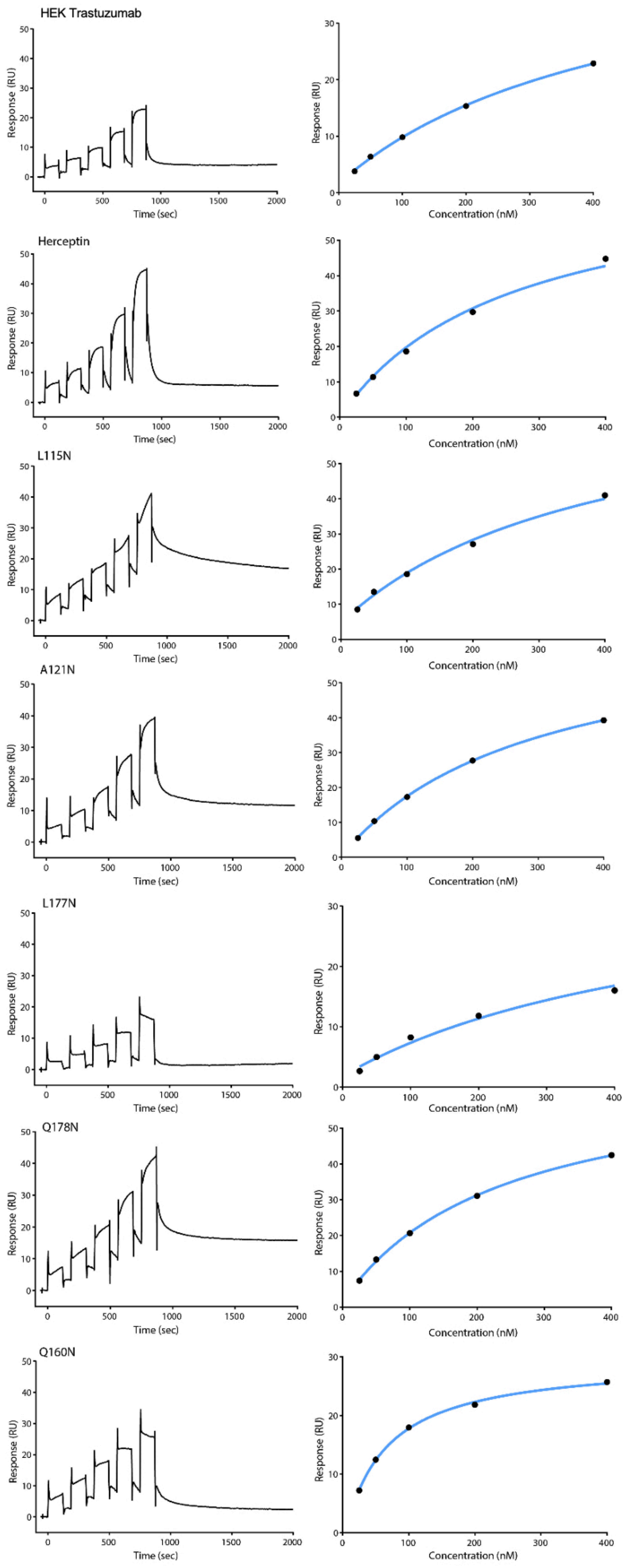

Figure S39. Left panel: SPR sensorgrams of single cycle kinetic assays of Tmab variants to surface captured FcγR3A. Right Panel: Fits to equilibrium responses from the sensorgrams.
